# Drawing from the Old‐The First Ever Sultone as Electrolyte Additive in High‐Voltage NMC811 || AG+SiOx Multilayer Pouch Cells

**DOI:** 10.1002/smll.202507089

**Published:** 2025-08-25

**Authors:** Matthias Weiling, Christian‐Timo Lechtenfeld, Silvan Stuckenberg, Felix Pfeiffer, Jian‐Fen Wang, Sascha Nowak, Verena Küpers, Masoud Baghernejad

**Affiliations:** ^1^ Helmholtz‐Institute Münster, IMD‐4 Forschungszentrum Jülich GmbH Corrensstrasse 46 48149 Münster Germany; ^2^ MEET Battery Research Center Münster University of Münster Corrensstrasse 46 48149 Münster Germany

**Keywords:** 1,8‐Naphthosultone, lithium‐ion batteries, *operando* ATR‐FTIR spectroscopy, silicon, solid‐electrolyte interphase

## Abstract

The addition of a small amount of silicon to the anode material is a widely used approach to increase the energy density of lithium‐ion batteries (LIBs). However, its (de‐)lithiation leads to volume changes, resulting in structural degradation and the formation of an insufficient solid‐electrolyte interphase (SEI), limiting the cycle life and electrochemical performance. Therefore, the formation of an effective SEI is imperative to overcome these challenges. Sulfur‐containing electrolyte additives are garnering attention due to their abundant supply and advantageous chemistry in LIBs. With 1,8‐naphthosultone (1,8‐NS) as an electrolyte additive, a notably enhanced electrochemical performance in high‐voltage NMC811 || artificial graphite(AG) + 20 % SiO*_x_* cells is observed. Employing advanced spectrometric and spectroscopic characterization techniques, complemented with theoretical calculations, the degradation products and pathways of 1,8‐NS in the cell are elucidated. This includes 1,8‐NS reduction, sultone ring opening, and chemical degradation with electrolyte solvent degradation products. The formation of these products is traced back to the SiO*_x_* anode, where an effective, layered SEI with various 1,8‐NS degradation products is formed. This SEI is suggested to exhibit improved mechanical and electrochemical parameters, resulting in the observed improvement of the electrochemical performance of the cells.

## Graphical abstract

Forming and understanding effective interphases is crucial to enabling next‐generation active materials for lithium‐ion batteries. The beneficial effect of 1,8‐naphthosultone as an electrolyte additive for interphase formation to increase the electrochemical performance of high‐voltage NMC811 || AG+SiO*_x_* pouch cells is presented. Interphase formation and electrolyte aging elucidation with advanced techniques allow the postulation of putative working mechanisms.

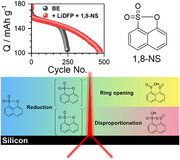

## 1 Introduction

Increasing the specific energy of lithium‐ion batteries (LIBs) is imperative to meet the rapidly growing demand for battery energy storage systems.^[^ ^1^, ^2^, ^3^ ^]^ The most straightforward approaches to achieving this objective are increasing the operating voltage of the LIB cells and enhancing the specific capacity of the positive and negative electrodes.^[^ ^4^, ^5^, ^6^, ^7^ ^]^ On the anode side, silicon is the most promising next‐generation active material, as the specific capacity of Li_15_Si_4_ is nearly ten times higher than that of state‐of‐the‐art graphite (LiC_6_).^[^ ^8^, ^9^, ^10^, ^11^ ^]^ However, severe volume changes during (de‐)lithiation pose a challenge, leading to particle pulverization and continuous active lithium and electrolyte consumption by ongoing (re‐)formation of the solid electrolyte interphase (SEI).^[^ ^12^, ^13^ ^]^ Consequently, a practical approach entails the incorporation of small fractions of understoichiometric silicon oxide in combination with graphite to buffer volume expansion.^[^ ^11^, ^14^, ^15^, ^16^, ^17^ ^]^ Although SiO*_x_* offers a lower specific capacity and reversible capacity than Si, the incorporation of 20 % SiO*_x_* already doubles the specific capacity of the anode.^[^ ^8^, ^11^ ^]^ Nevertheless, the electrochemical performance of the SiO*_x_*‐containing anode is predominantly influenced by its composition and SEI structure.^[^ ^18^, ^19^ ^]^ Analogous to state‐of‐the‐art LIBs, the ideal SEI on SiO*_x_*‐containing electrodes should facilitate rapid lithium ion transport while maintaining electrical insulation to avert continuous electrolyte degradation. Similarly, high mechanical and chemical stability is crucial to prevent particle cracking, structural and compositional degradation, and detachment of the formed interphase. Additionally, high flexibility is necessary to accommodate large volume changes.^[^ ^20^ ^]^ Consequently, the formation of artificial SEIs with the desired properties is necessary.

A cost‐effective and easily scalable solution to form more effective SEIs is the optimization of the electrolyte formulation. Apart from the solvents and the Li conducting salt, film‐forming electrolyte additives, such as vinylene carbonate (VC) or fluoroethylene carbonate (FEC), are widely used in LIBs to tailor the SEI even further.^[^ ^20^, ^21^ ^]^ For Si‐based batteries, in particular, sulfur‐containing electrolyte additives, such as PES (prop‐1‐ene‐1,3‐sultone) or DTD (1,3,2‐dioxathiolane‐2,2‐dioxide), have gained attention due to multiple reasons. For instance, sulfur‐containing electrolyte additives are reduced before their carbon‐containing counterparts. The facilitated reduction is due to a lower lowest unoccupied molecular orbital (LUMO) with sulfur. In addition, sulfur is abundant, easily accessible, and offers a wide variety of chemistries.^[^ ^22^, ^23^ ^]^ Embedded in the interphase, sulfur is usually present in combination with oxygen, *e.g*., in the form of sulfate or sulfone, enabling a high lithium ion conductivity.^[^ ^8^, ^22^, ^23^, ^24^, ^25^ ^]^

In this study, 1,8‐naphthosultone (1,8‐NS), the first compound classified as a “sultone” in 1888, is used as an electrolyte additive for SEI formation.^[^ ^26^ ^]^ This molecular structure also contains an aromatic naphthalene backbone, further lowering the LUMO and facilitating its reduction. Additionally, aromatic backbones were found beneficial in previous studies, as these are suggested to increase the chemical stability of the SEI.^[^ ^8^, ^24^, ^27^, ^28^ ^]^ For instance, Jankowski *et al.* suggest that the presence of an aromatic ring provides an improved adhesion to the graphite electrode.^[^ ^28^ ^]^ A beneficial impact of 1,8‐NS as an electrolyte additive on the electrochemical performance of LCO || AG (4.2–3.0 V) cells was already shown by Han *et al.* It is reported that 1,8‐NS leads to reduced internal resistance and increased discharge capacity for 35 cycles, compared to cells containing the baseline electrolyte (1 m LiPF_6_ in EC/EMC (3:7, by weight)).^[^ ^29^, ^30^ ^]^ Through the utilization of cyclic voltammetry, electrochemical impedance spectroscopy, and X‐ray photoelectron spectroscopy, the formation of a thin SEI film on the graphite surface was identified, comprising Li_2_S, R‐SO_2_Li, and R‐SO_3_Li as degradation products of 1,8‐NS.^[^ ^29^ ^]^ However, extended galvanostatic charge/discharge cycling and interphase and electrolyte characterization investigations are necessary to understand the working mechanisms of 1,8‐NS and the SEI formed by 1,8‐NS. In particular, it is imperative to characterize interphases *operando* or *in situ*, rather than *ex situ* or *post mortem*. This approach would facilitate the investigation of the formed interphases under real working conditions, as it mitigates sample contamination and chemical changes during preparation and transfer, thereby preserving the chemical environment and capturing reactive intermediate species.^[^ ^31^, ^32^, ^33^, ^34^, ^35^ ^]^

This study examines the impact of 1,8‐NS on the electrochemical performance of high‐voltage (4.5–2.8 V) NMC811 || AG+20 % SiO*_x_* multilayer pouch cells, electrolyte aging, and interphase formation. With an optimized concentration of 1,8‐NS, a substantial enhancement of the electrochemical performance of the cells is observed. The combination of 1,8‐NS with lithium difluorophosphate (LiDFP), a LiPF_6_ degradation product that scavenges transition metals to prevent rollover failure, further improves the electrochemical performance.^[^ ^36^, ^37^ ^]^ In comparison to cells with the baseline electrolyte, cells with both additives exhibit a cycle life enhancement until cell failure that exceeds +130%. Consequently, the energy storage capacity of the cells during their operational lifetime is enhanced by +50%. Furthermore, a comparative analysis involving electrolytes comprising state‐of‐the‐art and comparable electrolyte additives, including VC, FEC, DTD, and 2‐sulfobenzoic acid anhydride (2‐SBA), reveals the superior electrochemical performance of cells containing 1,8‐NS. The various degradation products in the electrochemically aged electrolyte and the corresponding degradation mechanism of 1,8‐NS in the cells are investigated by gas chromatography‐mass spectrometry (GC‐MS) and ion chromatography conductivity detection MS (IC‐CD‐MS). These degradation products are traced back to the evolving interphase on SiO*_x_* using *operando* attenuated total reflection Fourier‐Transform Infrared (ATR‐FTIR) spectroscopy. The chemical structure of the SEI is elucidated *via* simultaneous *operando* depth profiling. Additionally, the morphology and composition of the interphases on graphite compared to SiO*_x_* are characterized through scanning electron microscopy (SEM) and energy‐dispersive X‐ray spectroscopy (EDX).

## 2 Results and Discussion

### 2.1 Influence of 1,8‐NS on the Electrochemical Performance of High‐Voltage NMC811 || AG+20 % SiO_*x*_ Multilayer Pouch Cells

**Figure 1:**
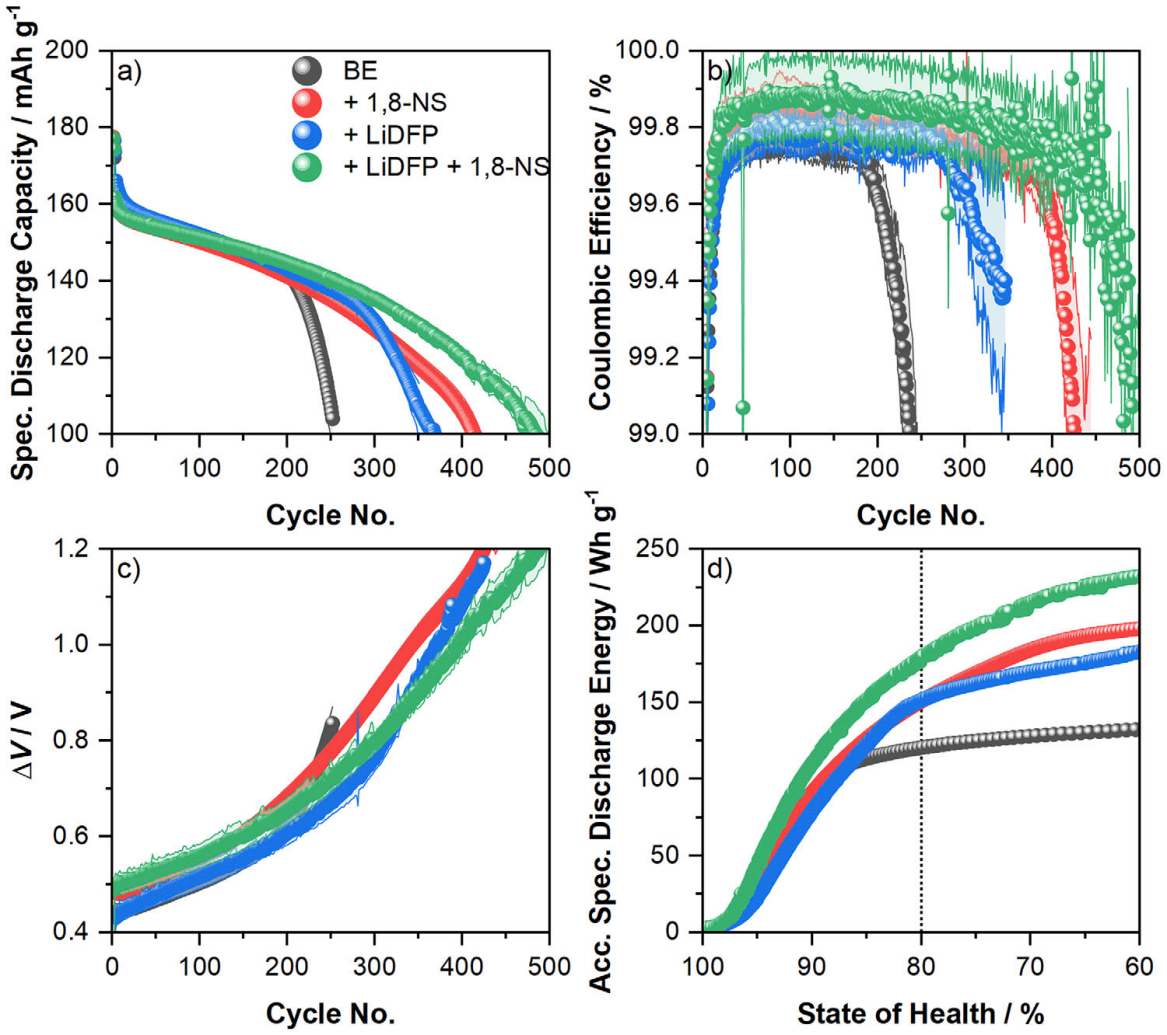
a) Specific discharge capacities, b) Coulombic efficiencies, c) Δ*V* *vs*. cycle number, and d) accumulated specific discharge energy *vs*. state of health of NMC811‖AG+20 % SiO_*x*_ multilayer pouch cells with 1.00 m LiPF_6_ in EC/EMC (3:7, by weight) as baseline electrolyte (BE), BE with 0.1 m 1,8‐naphthosultone (1,8‐NS), BE with 0.1 m lithium difluorophosphate (LiDFP), and BE with each 0.1 m as electrolyte additives. The CEs in the first cycle are 77.87 % ± 0.08 % for cells with BE, 77.63 % ± 0.06 % (BE+1,8‐NS), 78.58 % ± 0.03 % (BE+LiDFP), and 77.9 % ± 0.2 % (BE+LiDFP+1,8‐NS). Δ*V* is calculated as the difference between mean charge and mean discharge voltage.

The influence of an optimized concentration of 1,8‐NS as an electrolyte additive on the electrochemical performance of NMC811‖AG+20 % SiO*_x_* multilayer pouch cells is investigated by galvanostatic charge/discharge cycling, and the results are shown in **Figure** **1** with the corresponding errors in **Table** **1**. Further characterization of the electrochemical performance of varying additive concentrations, compared to 2‐SBA, DTD, FEC, and VC, is elucidated in the (Figures S1–S3 and Table S1, Supporting Information).

**Table 1:** Summary of the analysis results of the electrochemical performance of NMC811‖AG+20 % SiO_*x*_ multilayer pouch cells with 1.00 m LiPF_6_ in EC/EMC (3:7, by weight) as baseline electrolyte (BE), BE with 0.05 m 1,8‐Naphthosultone (1,8‐NS), BE with 0.1 m lithium difluorophosphate (LiDFP), and BE with each 0.1 m as electrolyte additives. Accumulated specific discharge energy (ASDE) is given at 80 % SoH, and the initial Δ*V* refers to the first cycle after formation at 1C. The percentage change compared to cells with BE is given in brackets.

Electrolyte Variation	Cycles Until 80 % SoH	Cycles Until Cell Failure	ASDE / Wh g^−1^	Mean CE / %	Initial Δ*V* / mV
BE	226 ± 1	195 ± 5	120 ± 1	99.75 ± 0.05	440 ± 20
+ 1,8‐NS	290 ± 5 (+31 %)	385 ± 5 (+97 %)	150 ± 2 (+25 %)	99.8 ± 0.1	486 ± 3
+ LiDFP	287 ± 3 (+26 %)	280 ± 5 (+44 %)	150 ± 3 (+25 %)	99.78 ± 0.05	430 ± 20
+ LiDFP + 1,8‐NS	351 ± 5 (+55 %)	460 ± 5 (+136 %)	180 ± 2 (+50 %)	99.9 ± 0.1	500 ± 20

In the case of reference cells containing the baseline electrolyte (BE, 1 m LiPF_6_ in EC/EMC, 3:7 by weight), a state of health (SoH) of 80 % was reached after 226 charge/discharge cycles. A SoH of cells lower than 80 % is generally regarded as the end‐of‐life for their respective first‐life applications. By introducing 1,8‐NS and LiDFP to the BE, the cells demonstrated a prolonged cycle life. After 295 cycles, the cells with 1,8‐NS attained 80 % SoH, marking a +31 % improvement. Similarly, the cells with LiDFP reached 80 % SoH with 287 cycles, exhibiting a +26 % enhancement. A more substantial enhancement was observed with the combination of both additives (BE + LiDFP + 1,8‐NS), resulting in a cycle life of 351 cycles (+55 %) until 80 % SoH.

Conversely, cells containing BE failed after 195 cycles and at ≈90 % SoH, as evidenced by a rapid decline in Coulombic Efficiency (CE) (Figure 1b). In contrast, the cells containing either 1,8‐NS (385 cycles, +97 %), LiDFP (280 cycles, +44 %), or both electrolyte additives (460 cycles, +136 %) exhibited a failure point below 70 % SoH. Furthermore, the CEs of the cells containing both electrolyte additives (99.9 %), BE + 1,8‐NS (99.8 %), or BE + LiDFP (99.78 %) during ongoing charge/discharge cycling were generally higher than the CEs of cells with BE (99.75 %) alone.

The incorporation of 1,8‐NS resulted in an increase of the internal resistance, as evidenced by an increase in the Δ*V* (Figure 1c, difference of mean charge and mean discharge voltage) during the initial charge/discharge cycles of the cells (BE + 1,8‐NS: 486 mV; BE + LiDFP + 1,8‐NS: 500 mV). This observation is in contrast to the cells that lacked 1,8‐NS, which exhibited a lower internal resistance (BE: 440 mV and BE + LiDFP: 430 mV). Conversely, an increasing Δ*V* is evident at elevated concentrations with increasing concentrations of 1,8‐NS above 0.1 m, as demonstrated in Figure S1 (Supporting Information). Notably, a decline in resistance was not detected in the initial cycles in the study by Han *et al.* Instead, a reduced internal resistance of the cells from ≈80 mΩ with BE to ≈40 mΩ with BE with 1 wt.% (≈0.06 m) 1,8‐NS was observed, leading to better lithium ion migration through the 1,8‐NS‐formed SEI on the graphite anode.^[^ ^29^ ^]^ The contrasting observations regarding the cell resistance may be attributable to the utilization of different anode materials, which notably impact the anode's electrochemical and chemical behavior. The higher cell resistance in this study may be associated with the development of a more resistive SEI on SiO*_x_* in the presence of 1,8‐NS, compared to the SEI formed on SiO*_x_* lacking 1,8‐NS and those formed on graphite. The disparities in SEI composition on SiO*_x_* compared to graphite will be discussed in more detail later. Notably, the generally higher resistive SEI does not appear to be detrimental to the overall electrochemical performance of the cells, as evidenced by maintaining unchanged specific discharge capacities and achieving longer cycle life.

The analysis of the accumulated specific discharge energy *vs*. SoH is a straightforward method for making fair comparisons between different cell variations. This is because it accounts for different cycle lives, capacities, and cell voltages. Until 80 % SoH, 120 Wh g^−1^ of accumulated specific discharge energy can be drawn from the cells containing BE (Figure 1d). In comparison, cells containing electrolyte additives exhibited higher accumulated specific discharge energies of 156, 150, and 180 Wh g^−1^ for cells with BE + 1,8‐NS (+25 %), BE + LiDFP (+25 %), and BE + LiDFP + 1,8‐NS (+50 %), respectively.

Consequently, high‐voltage NMC811‖AG+20 % SiO*_x_* cells with BE + LiDFP + 1,8‐NS exhibited superior electrochemical performance in terms of prolonged cycle life, reduced irreversible reactions, and an enhanced energy‐to‐lifetime ratio in comparison to cells with BE. To elucidate the underlying mechanisms responsible for the improved electrochemical performance of cells with 1,8‐NS, the electrolyte degradation products and the SEI structure were characterized using IC‐CD‐MS and *operando* ATR‐FTIR spectroscopy. The obtained results are complemented with DFT calculations and GC‐MS analysis.

### 2.2 Investigation of 1,8‐NS Degradation Products

**Figure 2:**
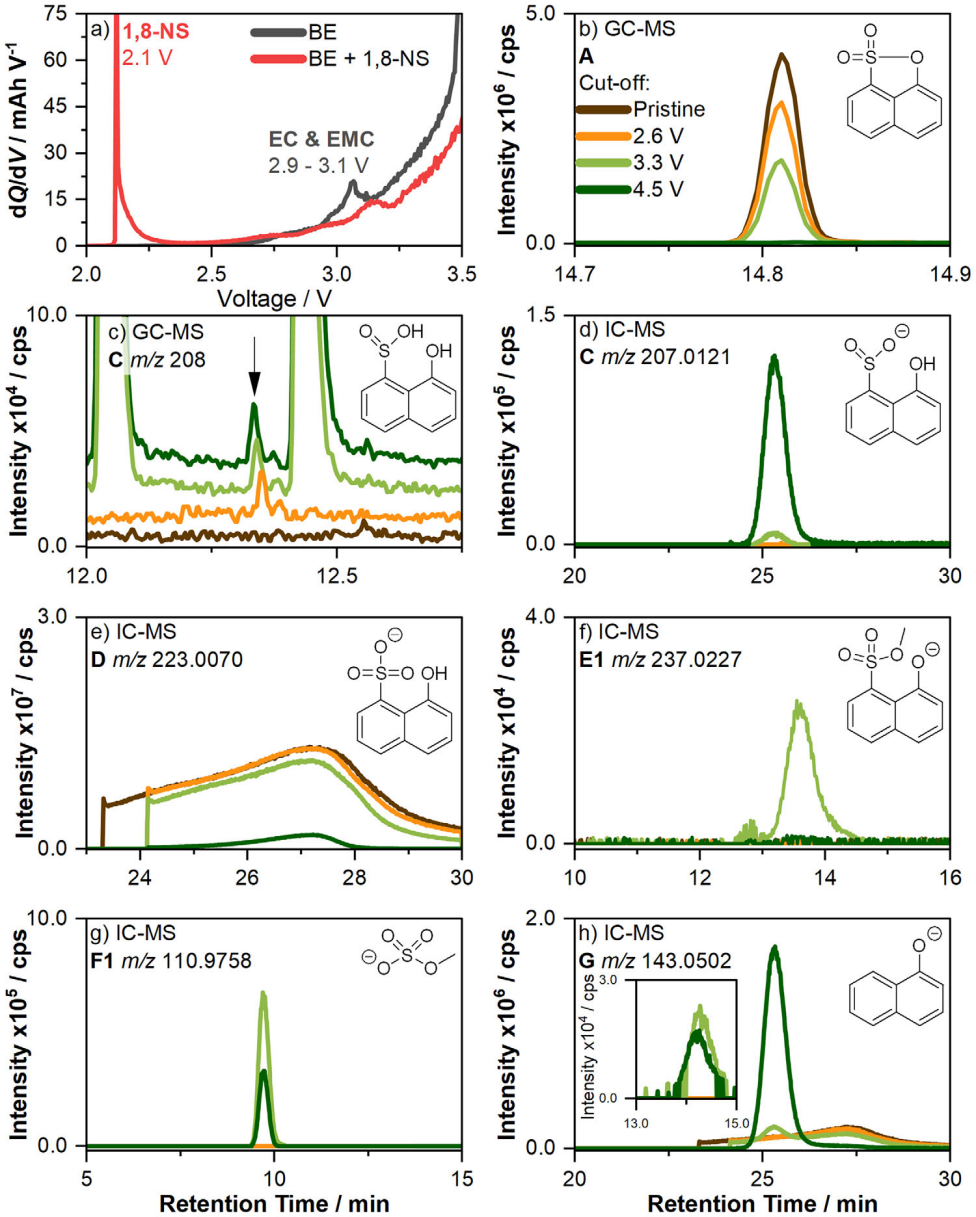
a) Enlarged d*Q*/d*V* graph in the range of 2.0 V to 3.5 V of the first charge in the first formation cycle of NMC811 || AG+20 % SiO_*x*_ cells with BE and BE + 0.1 m 1,8‐NS as electrolyte. The full d*Q*/d*V* graph is shown in Figure S4 (Supporting Information). Sections of the GC‐MS TICs of electrolyte samples representing b) 1,8‐NS and c) degradation product **C** *m*/*z* 208. The full GC‐MS chromatogram can be found in Figure S6 (Supporting Information). Sections of the IC‐CD‐MS EICs of degradation products d) **C** *m*/*z* 207.0121, e) **D** *m*/*z* 223.0070, f) **E1** *m*/*z* 237.0227, g) **F1** *m*/*z* 110.9758, and h) **G** *m*/*z* 143.0502. The EICs of **E2**, **E3**, **F2**, and **F3** are shown in Figure S9 (Supporting Information). The electrolyte samples were obtained from the pristine electrolyte and extracted electrolytes from cells charged to 2.6 V, 3.3 V, and 4.5 V.

In the d*Q*/d*V* analysis in **Figure** **2**a, an additional peak is identified at a cell voltage of 2.1 V in the voltammogram of the cells with 1,8‐NS, which can be attributed to the reduction of 1,8‐NS. The addition of 1,8‐NS to the electrolyte solution has been shown to suppress the broad peak at 2.9 V to 3.1 V, which is attributed to EC and EMC reductive degradation. This peak is observed to shift toward higher cell voltages. This observation suggests a reduced rate of EC and EMC reductive degradation and a marginally higher overvoltage for this process in the presence of 1,8‐NS. Furthermore, as shown in the full d*Q*/d*V* curve of the initial charge (Figure S4, Supporting Information), the SiO*_x_* and AG lithiation peaks in the case of BE+1,8‐NS are shifted to higher voltages by (70 ± 10) mV and (40 ± 20) mV compared to BE, respectively. This observation indicates the formation of a more resistive SEI with 1,8‐NS, as evidenced by the overall higher Δ*V* (Figure 1c) in the presence of 1,8‐NS. The unequal peak shift may also suggest a more resistive SEI on SiO*_x_* compared to graphite.

**Figure 3:**
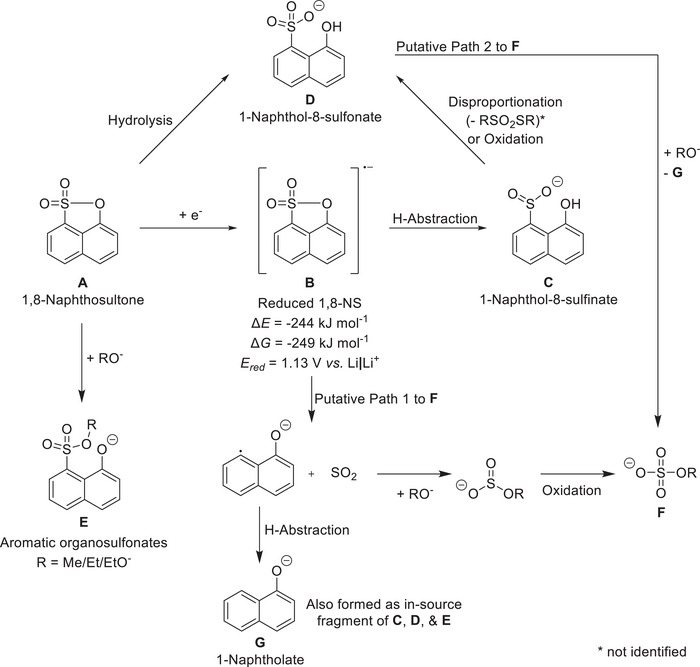
Putative degradation overview of 1,8‐NS (**A**) reduction to reduced 1,8‐NS (**B**), further reacting to 1‐naphthol‐8‐sulfinate (**C**), putative chemical reaction of **A** to 1‐naphthol‐8‐sulfonate (**D**) by hydrolysis in the IC column, and addition of lithium alkoxides to **A** resulting in aromatic organosulfonates (**E**). **C** may further be oxidized or disproportionate to **D** and RSO_2_SR (not identified). Aliphatic organosulfates (**F**) are assumed to arise from either SO_2_ elimination from **B**, followed by lithium alkoxide addition to SO_2_ and subsequent oxidation, or addition of alkoxides to **D** and elimination of **G**. 1‐Naphtholate (**G**) is identified as in‐source fragment of **C**, **D**, and **E** and might be formed by hydrogen abstraction after the SO_2_ cleavage. Note that due to the complexities and differences of the battery, GC, and IC matrices, the reactions are not balanced and the molecular structures might be present (de‐)protonated or coordinated with lithium cations or electrolyte solvent molecules.

To establish a correlation between the 1,8‐NS (**A**, see **Figure** **3**) reduction voltage and a chemical structure, DFT calculations were conducted. The reduction of 1,8‐NS at the anode is calculated to occur at a potential of 1.1 V *vs*. Li|Li^+^ (Figure 3). This finding is in alignment with the previously experimentally determined reduction potential of 1.1 V *vs*. Li|Li^+^.^[^ ^29^ ^]^ Furthermore, DFT calculations were utilized to estimate the bond orders in the reduced 1,8‐NS (**B**) molecule, which allow a prediction of the bond strengths in the intermediate structure. The subsequent ring opening of the sulfone ring of the reduced 1,8‐NS (**B**) is predicted to occur preferably at the S─O position, instead of the C─O or C─S position (see Figure S5, Supporting Information). The respective bond order in the reduced 1,8‐NS structure is calculated to be 0.68 (S─O), which is lower compared to 0.93 (C─O) and 0.97 (C─S). Additionally, the S‐O bond length exceeds the typical value, attributable to the rigid naphthalene backbone.^[^ ^38^ ^]^

The degradation products of 1,8‐NS in the electrolyte are determined experimentally *via* *ex situ* GC‐MS and IC‐CD‐MS. It is important to note that in this study, both methods lack the capacity to quantitatively compare different molecules since the ionization rates for different molecules are not considered. Nevertheless, relative quantitative changes of the same molecule are indicated. In the samples of the extracted electrolytes, the area between 17.5 min to ≈24.0 min is cut out and not injected into the MS, as the PF_6_ anion would cause mass overloading.

Upon the initial formation charge, cells were stopped at upper cutoff voltages following 1,8‐NS reduction (2.6 V), electrolyte solvent reduction (3.3 V), and a full charge (4.5 V). The resulting total ion chromatograms (TIC, for GC‐MS) and extracted ion chromatograms (EIC, for IC‐CD‐MS) are displayed in Figure 2b–f and compared to the ones obtained from the pristine electrolyte sample. It is noteworthy that due to the disparate chemical environments present in the GC‐MS and IC‐CD‐MS analysis in comparison to the electrolyte and interphase environments, the protonation of the degradation products in the cells remains uncertain.

The depletion of 1,8‐NS (**A**, 14.81 min) in the electrolyte with increasing cut‐off potentials is demonstrated by the decreasing intensities in the GC‐MS‐TICs until the limit of detection is almost reached when the cells are charged to 4.5 V (Figure 2b). Thus, it is hypothesized that the majority of 1,8‐NS (**A**) is gradually consumed during the initial formation charge, either chemically or electrochemically. The consumption is split into three parts. Initially, the consumption up to 2.6 V is attributed to the reduction of 1,8‐NS (**A**) on the anode, as evidenced by d*Q*/d*V* analysis. The observation of a still relatively high TIC intensity in the sample obtained at 2.6 V suggests the reduction of only a small amount of 1,8‐NS. This might indicate the formation of a passivating layer comprising 1,8‐NS degradation products, which hinders the ongoing and complete reduction of 1,8‐NS. Second, the consumption of 1,8‐NS (**A**) between 2.6 V and 3.3 V might be attributed to chemical degradation with electrolyte solvent degradation products, as the electrolyte solvents EC and EMC are reduced in this voltage range. Third, the consumption of 1,8‐NS (**A**) between 3.3 V and 4.5 V might be attributed to the ongoing reduction on the anode surface or chemical reaction with other degradation products, caused by the freshly evolving anode surface from volume expansion. To illuminate the various degradation pathways, putative degradation products, and mechanisms, a detailed discussion will be presented in the subsequent sections.

As indicated by DFT calculation, the predominant reduction product of 1,8‐NS is the S─O ring‐opened product (**C**, 8‐hydroxynaphthalene‐1‐sulfinate, *m*/*z* 208, 12.34 min), which is corroborated by GC‐MS analysis (Figure 2c) in the samples obtained at 2.6 V, 3.3 V, and 4.5 V. This finding lends further support to the aforementioned assumption of putative reductive degradation. However, the presence of **C** was not detected in the sample obtained at 2.6 V *via* IC‐CD‐MS (Figure 2d, *m*/*z* 207.0121, 25.33 min). Conversely, **C** was identified in the sample obtained at 3.3 V and with the highest intensity in the sample obtained at 4.5 V. This discrepancy could be attributed to the varying prerequisites for the distinct techniques employed. Specifically, for IC, **C** can exist as a sulfinate, whereas for GC, **C** must exist as a volatile sulfinic acid. Given the likelihood of **C** forming as a sulfinate species, its protonation is less probable in the interphase and electrolyte environment due to the presence of other species. For instance, the acidity of aromatic sulfinic species is generally stronger than that of the corresponding carboxylic acids.^[^ ^39^ ^]^ It is hypothesized that the intensity increase of the samples investigated with IC‐CD‐MS indicates the reduction of only a small fraction of the 1,8‐NS up to 2.6 V, leading to the formation of a thin layer on the anode, preventing more reductive decomposition. Furthermore, volume expansion with lithiation of the SiO*_x_* above 2.6 V leads to the evolution of “fresh” anode surface, which in turn results in additional reduction of 1,8‐NS to **C**. This process might explain the ongoing depletion of 1,8‐NS (**A**) upon charging, as observed *via* GC‐MS (Figure 2b).

Theoretically, *m*/*z* 208 (GC‐MS) or *m*/*z* 207.0121 (IC‐CD‐MS) could also correspond to the structural isomer naphthalene‐1‐sulfonate. However, this is unlikely due to the ring‐opening preferences, as discussed above. In case of *m*/*z* 208 (GC‐MS), the experimentally obtained fragmentation pattern does not correspond with the fragmentation pattern of the structural isomer naphthalene‐1‐sulfonate given in the literature (Figure S8, Supporting Information). In case of *m*/*z* 207.0121(IC‐CD‐MS), 1‐naphtholate (**G**, Figure 2h, *m*/*z* 143.020, 25.33 min) was detected at the same retention time. Furthermore, **G** was identified in the electrolyte sample obtained from the cells charged to 3.3 V and with a maximum in the electrolyte sample obtained from the cells charged to 4.5 V. It is assumed that **G** is formed from the sulfinate (**C**) by the cleavage of SO_2_ due to in‐source fragmentation during the ionization.^[^ ^40^ ^]^ To determine the actual retention time of **G**, 1‐naphthol was measured as a reference component in an additional experiment (Figure S7b, Supporting Information). As a result, a retention time of 20.10 min was found for **G**. As **G** cannot be formed from naphthalene‐1‐sulfonate, it is indicated that naphthalene‐1‐sulfonate is not formed. Consequently, it is indicated that *m*/*z* 207.0121 is derived from 1‐naphthol‐8‐sulfonate (**C**) and not 1‐naphthalenesulfonate. As a potential subsequent reaction, aromatic sulfinic acids (such as **C**) are reported to disproportionate to sulfonic acids (RSO_3_H, such as **D**) and a thiosulfonate (RSO_2_SR), however, the dimer was not identified in this study. Alternatively, oxidation of **C** within the cell might result in the formation of **D**. This topic will be discussed further below.^[^ ^41^, ^42^ ^]^

The reduction of EC and EMC at cell voltages <3.3 V is known to result in the formation of lithium alkoxides, such as methanolate and ethanolate. These alkoxides have been identified as the initiating agents for the continuous chemical degradation of electrolyte compounds.^[^ ^43^, ^44^ ^]^ For instance, these reactions result in transesterification with products such as dimethyl and diethyl carbonate (DMC, DEC), and carbonate oligomers (OHCs), like dimethyl, ethyl methyl, and diethyl 2,5‐dioxahexane dicarboxylate (DMDOHC, EMDOHC, DEDOHC), as shown in Figure S6 (Supporting Information).^[^ ^8^, ^44^, ^45^ ^]^ In this case, this also leads to the formation of 1,8‐NS chemical degradation products. The presence of these products is observed in the sample obtained at a cell voltage of 3.3 V. As shown, lithium alkoxides in combination with **A** lead to the formation of aromatic organosulfonates (**E**), including methyl (**E1**, *m*/*z* 237.0227, 13.55 min, Figure 2f), ethyl (**E2**, *m*/*z* 251.0383, 14.25 min, Figure S9a, Supporting Information), and ethylene glycol (**E3**, *m*/*z* 267.0332, 15.10 min, Figure S9b, Supporting Information).^[^ ^46^ ^]^ Similar to **C**, **G** (14.3 min) is also identified as an in‐source fragment of **E**. Additionally, **G** (Figure 2h, 27.25 min) is identified as an in‐source fragment of the 1‐naphthol‐8‐sulfonate (**D**, Figure 2e, *m*/*z* 233.0070, 27.16 min). As previously mentioned, **D** can be formed by disproportionation of **C**, by hydrolysis with residual moisture of 1,8‐NS, or both.^[^ ^46^ ^]^ Furthermore, the relatively high intensity might be caused by the hydrolysis of pristine 1,8‐NS in the electrolyte in the IC column, using an aqueous medium.

Furthermore, aliphatic organosulfates, specifically methyl sulfate (**F1**, *m*/*z* 110.9758, 9.69 min, Figure 2g), ethyl sulfate (**F2**, *m*/*z* 124.9914, 9.74 min, Figure S9c, Supporting Information), and ethylene glycol sulfate (**F3**, *m*/*z* 140.9863, 9.10 min, Figure S9d, Supporting Information), are identified. These are observed with maximum intensity in the samples obtained at 3.3 V. In the electrolyte sample from the cells charged to 4.5 V, the intensity of **F1** decreases to half of the intensity of the sample obtained at 3.3 V. In the following paragraphs, three possible origin and formation mechanism of these components are discussed.

First, in‐source fragmentation is not likely to result in the addition of aliphatic rests to sulfate or the addition of alkoxylates to sulfonates, accompanied by C─S bond cleavage. This assertion is further substantiated by the absence of **G** as in‐source fragment. Second, based on the literature, the feasibility of the formation of **F** in this system during cell operation is debatable, as desulfonylation typically occurs radical‐mediated and requires a rearrangement or catalysis to remove sulfur dioxide from sulfonate.^[^ ^47^, ^48^, ^49^, ^50^ ^]^ To form **F** from SO_2_, the addition of alkoxides to SO_2_ and oxidation of the resulting RSO_3_^−^ at the cathode at a cell voltage of 3.3 V would need to occur (Putative Path 1 to **F**, see Figure 3). The probability of the reaction following this putative reaction path is supported by literature showing the reactions of sulfur dioxide with alkoxylates to RSO_3_^−^ and the oxidation of SO_3_^−^ to SO_4_^−^, although under different conditions.^[^ ^47^, ^48^, ^49^, ^50^, ^51^, ^52^ ^]^ Third, addition of alkoxylate to **D** and elimination of **G** (after hydrogen abstraction) might lead to the formation of **F** (Putative Path 2 to **F**). To elucidate the feasibility of this reaction path for the formation of **F** in this system, an additional experiment was conducted. Here, lithium methoxide was added to **D** in DMC in a glovebox, and the solution was stirred for two days at 20 °C. The IC‐CD‐MS analysis of the solution revealed the formation of **F1** (Figure S7a, Supporting Information, *m*/*z* 110.9775, 8.85 min), albeit in a relatively low quantity, thereby substantiating that a chemical cleavage of sulfate from the aromatic backbone is indeed a feasible reaction path. This finding is counterintuitive, as the C─S bond is usually considered relatively stable (discussed above and shown in Figure S5, Supporting Information). Nevertheless, the cleavage of the C─S bond of different sultones used as electrolyte additives is reported in several studies.^[^ ^22^ ^]^ Note that the majority of the studies postulate Li_2_SO_3_, some studies postulate Li_2_SO_4_ as product. This is relatively ambiguous, as the peak ≈169 eV in the reported XPS spectra is assigned to both Li_2_SO_4_^[^ ^53^, ^54^ ^]^ and Li_2_SO_3_^[^ ^23^, ^55^, ^56^, ^57^, ^58^, ^59^, ^60^ ^]^ (also sometimes 167 eV – 168 eV). As the literature on the mechanistic understanding of the two putative paths is scarce, follow‐up studies are encouraged to investigate the (electro‐)chemical degradation leading to **F** to shed light on the preferred formation mechanism. Conversely, the addition of lithium methoxide to a solution of **A** in DMC resulted in the formation of **E1**. However, the subsequent formation of **F1** out of **E1** was not observed. Due to the retention time of **G** being in the cut‐out section, the formation of **G** was not directly observed. Nevertheless, the identification of the counterpart **F** as a degradation product in the cells suggests that the formation of **G** may also occur.

Consequently, an additional beneficial effect of 1,8‐NS (**A**) might be the scavenging of Lewis bases, such as water (reaction pathway **A** to **D**) or alkoxides (reaction pathway **A** to **E**). It is widely documented that Lewis bases can be detrimental to the lithium‐ion battery system, as they have been observed to accelerate electrolyte aging and interphase deterioration.^[^ ^61^, ^62^, ^63^ ^]^ This assertion is corroborated by the enhanced electrochemical performance of cells with 1,8‐NS, as previously discussed.

In conclusion, the nearly complete consumption of 1,8‐NS (**A**) in the initial formation charge is observed. The reduced 1,8‐NS (**B**) is found to degrade to the S─O ring opened sulfinate (**C**), as proven by **G** as an in‐source fragment. Furthermore, a putative subsequent reaction involves the chemical reaction of **A** with lithium alkoxides, resulting from electrolyte solvent degradation to aromatic organosulfonates (**E**). The sulfonate group‐containing degradation products (**D**) may undergo further reactions to aliphatic organosulfates (**F**) and 1‐naphtholate (**G**).

### 2.3 Interphase Analysis *via Operando* ATR‐FTIR Spectroscopy

Above, a putative degradation mechanism for 1,8‐NS was postulated employing *ex situ* methods sampling the electrochemically aged electrolyte. However, the reliability of the results can be compromised by aging effects during *ex situ* investigations. This includes reactions of unstable intermediate species during sample storage, sample contamination during preparation, and changes in the chemical environment, such as the aqueous medium in the IC column. Additionally, as the SEI determines the electrochemical performance of the cells to a large extent, the characterization of its structure and composition is crucial to substantiate the enhanced electrochemical performance. Thus, to further support the aforementioned assumptions and complement the *ex situ* analyses, the formation of the SEI in the presence of 1,8‐NS is investigated close to real working conditions employing *operando* ATR‐FTIR spectroscopy. For detailed information about the technical aspects of *operando* vibrational spectroscopy or this particular ATR‐FTIR spectroelectrochemical cell, the reader is referred to previous publications.^[^ ^8^, ^31^, ^64^ ^]^

To minimize the overall number of interfering bands in the IR spectrum, the electrolyte solvent DMC is used instead of EC/EMC in combination with LiBF_4_ instead of LiPF_6_ (BE2, see Figure S10, Supporting Information). The absence of EC has been shown to reduce or even prevent the amount of some electrolyte solvent degradation products, such as OHCs or (lithium‐)organo dicarbonates.^[^ ^65^, ^66^ ^]^ Simultaneously, methoxy groups from DMC degradation should still be present to resemble electrolyte degradation products as reactants with 1,8‐NS.^[^ ^43^, ^44^ ^]^

**Figure 4:**
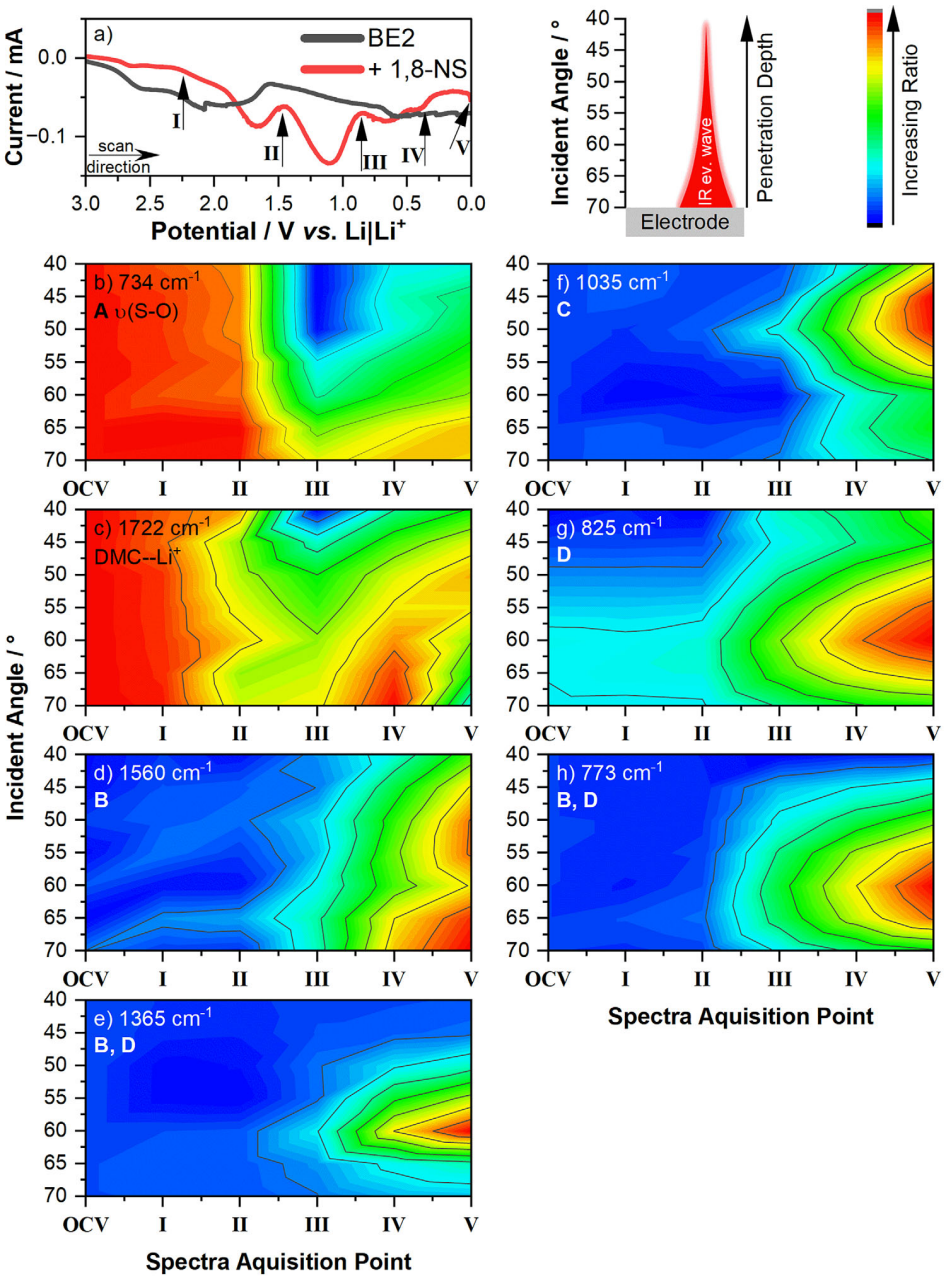
a) Current voltammogram of the Si || Li spectroelectrochemical cell with a lithium reference electrode and marker of the *operando* measurement points. b–h) heatmaps of the relative ratios of the bands ≈734 cm^−1^, 1722 cm^−1^, 1560 cm^−1^, 825 cm^−1^, 1365 cm^−1^, 1035 cm^−1^, and 773 cm^−1^. The ratios are calculated vs. the same band at OCV. Increasing ratios indicated by the given color scheme indicate increasing band intensities, compared to OCV, and *vice versa*.

A comparison of the voltammograms of the cells with the BE2 and BE2+1,8‐NS in the linear sweep voltage scans in **Figure** **4**a reveals the presence of an additional additive reduction peak at ≈1.1 V *vs*. Li|Li^+^. This observation is consistent with the previously mentioned reduction potentials of 1,8‐NS. The arrows indicate the positions at which the IR spectra were acquired (**I** to **V**). As this occurred during cell polarization, the spectra were acquired with a deviation of 0.1 V *vs*. Li|Li^+^ at the respective point. The heatmaps in Figure 4 are generated as ratios of the absorbance of the respective bands to the same band at OCV to account for the generally lower intensities at higher incident angles. The incident angle is inversely proportional to the penetration depth of the IR evanescent wave; therefore, spectra at a high incident angle represent sampling in the electrode vicinity, and vice versa. In the ensuing analysis, the upper SEI layer is defined as the SEI located farther from the electrode and closer to the bulk electrolyte (low incident angles), whereas the lower SEI refers to the SEI layer positioned closer to the electrode (high incident angles). It is important to note that minor alterations in the ratio may be attributable to the generally low signal intensities, particularly at higher incident angles (see Figure S11, Supporting Information). For reference, the spectra obtained from a cell without 1,8‐NS additive are shown in Figure S12 (Supporting Information), which displays the absence of bands assigned to 1,8‐NS degradation products. Theoretical IR spectra of putative degradation products in the interphase are calculated by DFT calculations (Tables S3 and S4, Supporting Information); however, due to a complex matrix and different chemical environments in the interphase compared to bulk electrolyte or pristine materials, the band assignments must be considered with care. Furthermore, IR spectra of 1,8‐NS, 1,8‐NS + H_2_O, 1,8‐NS + LiOMe, sodium 1‐naphthol‐8‐sulfonate, and sodium 1‐naphthol‐8‐sulfonate + LiOMe dissolved DMC are shown in Figure S13 (Supporting Information).

By comparison of the reference spectra of pure 1,8 and of 1,8‐NS in the electrolyte (Figure S14, Supporting Information) with DFT calculations of the IR spectrum (Table S2, Supporting Information), the S─O stretching vibration of pure 1,8‐NS is assigned to the band ≈734 cm^−1^ (*ν*(S─O)). As depicted in Figure 4b, the heatmap of the *ν*(S─O) band for pristine 1,8‐NS reveals the presence of 1,8‐NS (**A**) with comparable intensities across all incident angles at **OCV**, **I**, and **II**. This observation suggests that 1,8‐NS is uniformly distributed before the initial electrochemical processes occur. However, between **II** and **III**, a notable decrease in the ratio is observed, indicating a consumption of **A** due to its reduction. Conversely, between **III** and **V,** the ratios exhibit an increase at higher incident angles, suggesting the presence of **A** in the lower SEI, concurrent with lithiation of the SiO*_x_*. This trend is replicated in the bands ≈1190 cm^−1^ and 1164 cm^−1^, both assigned to *δ*_ip_(ring) + *ν*_s_(S═O) (Figure S15, Supporting Information).

The band ≈1722 cm^−1^ (Figure 4c) can be assigned to *ν*(C═O) of DMC coordinated with Li^+^. In a manner analogous to 1,8‐NS, the ratios suggest an even distribution of the electrolyte solution at **OCV**. It is shown that the ratios undergo a decline between **I** and **III**, an increase between **III** and **IV** at high incident angles, and a subsequent decrease between **IV** and **V**. It is hypothesized that the lithium ions are coordinately bonded to the reduced 1,8‐NS or a similar structure, rather than to DMC during the transition from **II** to **III**. This hypothesis is postulated to result in a decrease in intensity. The increasing intensities observed at high incident angles at **IV** are assumed to be attributable to the formation of a thin lithium‐rich layer on the polarized electrode. At **V**, the reduced intensity is attributed to the depletion of lithium ions in the SEI, resulting from lithium insertion into the Si electrode.

Additional bands ≈1560 cm^−1^, 1365 cm^−1^, 1035 cm^−1^, 825 cm^−1^, and 773 cm^−1^ become increasingly intense commencing from **III** (see heatmaps of the recorded IR spectra in Figure 4d–h). These bands were not detected in the investigation of the cell with the electrolyte without 1,8‐NS. This observation suggests that these bands may be indicative of degradation products of 1,8‐NS in the SEI. In subsequent paragraphs, the validation of the identified structures and proposed degradation pathways of 1,8‐NS (Figure 3) in the *operando* measurement is discussed. It should be noted that no reference components were available for compounds **B** and **C**. Consequently, DFT calculations were employed to predict the IR spectra of these molecules (see Tables S3 and S4, Supporting Information).

In accordance with the proposed reduction mechanism, the initial step entails the reduction of 1,8‐NS to **B**. The calculated IR spectrum of **B** indicates the presence of vibrational bands ≈1560 cm^−1^ (*ν*(C═C), Figure 4d), 1365 cm^−1^ (*ν*(C═C) + *δ*_ip_(C─H), Figure 4e), and 773 cm^−1^ (*δ*_oop_(C─H), Figure 4h) (Table S3, Supporting Information). A comparison of the DFT‐calculated IR vibrations with the recorded IR spectra reveals that the characteristic band ≈1560 cm^−1^ is found at increasing voltages. The presence of characteristic bands assigned to **B** in the IR spectra indicates that the reduced structure is relatively stable and does not directly further react to other degradation products. This increased stability for intermediate products of aromatic sulfur‐containing additives compared to their non‐aromatic counterparts was also observed in previous studies.^[^ ^23^, ^28^ ^]^ As shown in Figure 4d, the intensities of this band begin to appear from **II** to **III**, corresponding to the range of 1,8‐NS reduction. The observed increase in intensity in the heatmap is assumed to be due to the ongoing reduction of **A** to **B** and the subsequent accumulation of **B**. The angular resolution indicates the accumulation of **B** in the region between 65° and 70°, particularly at point **V**, which corresponds to the lower SEI in the electrode vicinity. The angular resolution of the bands ≈773 cm^−1^ and 1365 cm^−1^ indicates the highest ratio increases at 60°, thereby supporting the accumulation of **B** in the lower SEI. However, the heatmap for the band ≈1560 cm^−1^ at lower incident angles shows increased intensities, indicating the presence of **B** in the upper SEI.

Following the proposed mechanism (Figure 3), the subsequent step is the S─O ring opening from **B** to **C**. Through a comparison of the DFT calculations and the recorded reference IR spectra, the band ≈1035 cm^−1^ (*ν*(C═C), Figure 4f) is characteristic of **C**. This band exhibits increasing ratios between **III** and **IV**, rather than between **II** and **III**, which might indicate a somewhat delayed formation of **C** below 1 V *vs*. Li|Li^+^. Furthermore, the angular resolution shows increasing ratios at incident angles ≈45° to 50°, indicating the presence of **C** in the upper SEI and thus further away from the electrode. A comparison of the reference spectrum of sodium 1‐naphthol‐8‐sulfonate (**D**) in DMC (Figure S13, Supporting Information) and DFT calculations (Table S4, Supporting Information) exhibits bands ≈1365 cm^−1^ (*δ*_ip_(C─H)), 825 cm^−1^, and 773 cm^−1^ (*δ*_ip_(C─H)), with the band ≈825 cm^−1^ (Figure 4g) being characteristic for **D**. The angular resolution reveals notably increasing ratios of all three bands ≈60° at potentials above 1.5 V *vs*. Li|Li^+^. Therefore, it is indicated that similar to **B**,**D** is preferably located in the lower SEI on the SiO*_x_* surface.

As previously discussed, alkoxides from linear carbonates are necessary for the formation of aromatic organosulfonates (**E**), aliphatic organosulfates (**F**), and 1‐naphthol (**G**). Notably, the band ≈825 cm^−1^ (Figure 4f) is frequently assigned to *δ*(CO_3_) of lithium methyl carbonate (LMC), a prevalent DMC degradation byproduct. However, the recorded spectra in this study exhibit the absence of other LMC‐characteristic bands, suggesting that no quantitative DMC degradation occurs, thereby hindering the formation of methoxide or other DMC degradation byproducts.^[^ ^65^, ^66^, ^67^, ^68^ ^]^

In summary, the pristine 1,8‐NS (**A)** is found to be reduced at the Si electrode. The reduced 1,8‐NS (**B**) is preferably located in the lower SEI at the electrode, but also diffuses into the upper SEI with ongoing time. Furthermore, the sulfinate (**C**) product is found in the upper SEI, whereas the sulfonate (**D**) product is located in the lower SEI. It is hypothesized that these aromatic backbone‐containing products enhance the mechanical stability, flexibility, and ionic conductivity of the formed interphase on the anode.

### 2.4 Visual and X‐ray Analysis of the Interphase

**Figure 5:**
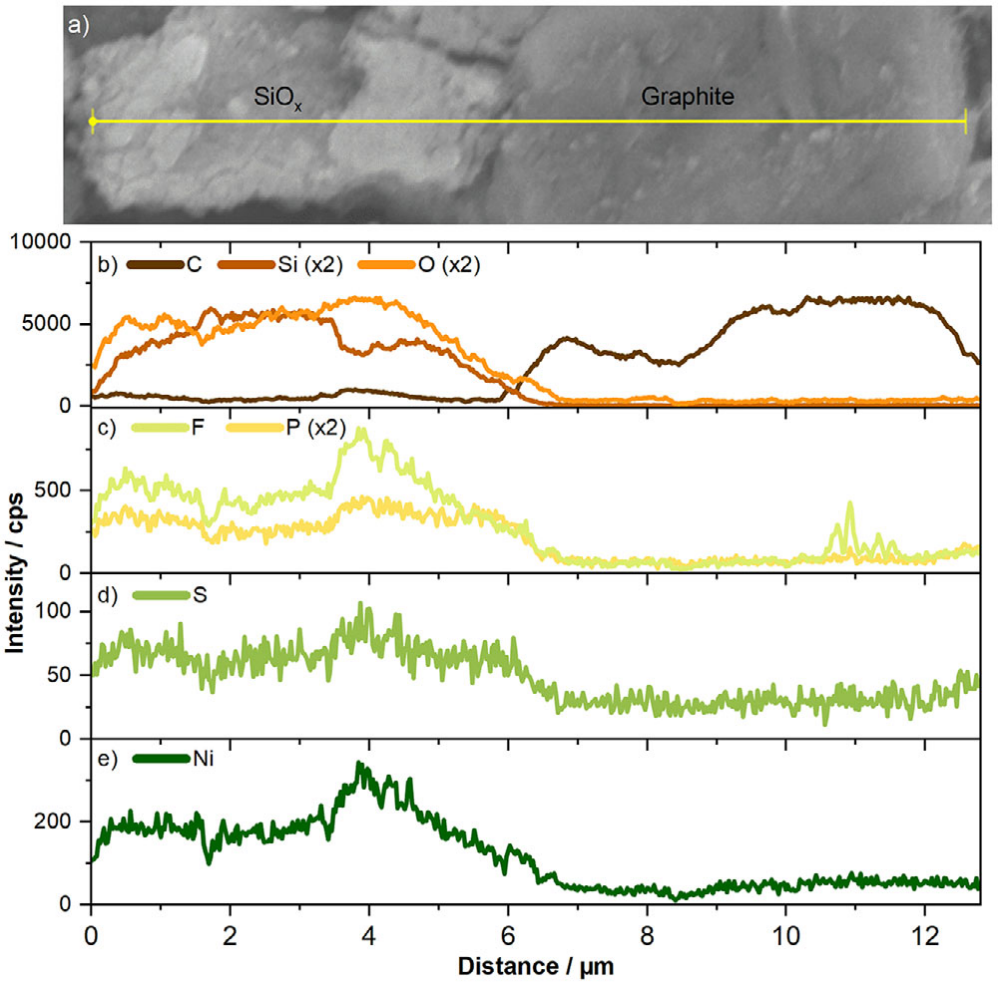
a) SEM image of the anode from NMC811‖ AG+20 % SiO_*x*_ multilayer pouch cells charge/discharge cycled to 150 cycles showing the EDX line scan on a SiO_*x*_ particle and an AG particle. EDX line scan intensities of b) C, Si (x2), O (x2), c) F, P (x2), d) S, and e) Ni on the particles shown in a). The electrodes were washed with DMC before the measurement.

While the investigation *via operando* ATR‐FTIR spectroscopy was performed on a SiO*_x_* electrode without graphite, a visual investigation through *post mortem* SEM reveals different surfaces of the graphite particles compared to the SiO*_x_* particles (**Figure** **5**a; Figure S16, Supporting Information). Images of an electrode that was charge/discharge cycled for 150 cycles indicate different interphase thicknesses on SiO*_x_* and graphite particles. EDX was utilized to perform line scans, which allowed for the characterization of the surface composition of the particles. Prior to the measurement, the anodes were washed with DMC, thus mostly the less soluble species of the SEI were left on the electrode.^[^ ^69^, ^70^ ^]^ The line scan of carbon, silicon, and oxygen (Figure 5b) unequivocally identifies the particles as SiO*_x_* (left) and graphite (right). The deposition or degradation of LiPF_6_ as an SEI component is the sole source of phosphorus and fluorine on the anode, with its intensity indicating the thickness of the SEI on the different particles. In Figure 5c a higher intensity of phosphorus ((160 ±70) cps) and fluorine ((580 ± 290) cps) is found on the SiO*_x_* particle compared to the graphite particle (P: (50 ± 40) cps; F: (80 ± 50) cps), indicating a thicker SEI on the SiO*_x_* particle.

A similar trend is observed in the sulfur analysis (Figure 5d), indicating the incorporation of 1,8‐NS degradation products with a low electrolyte solubility in the SEI, particularly on SiO*_x_*. In a second investigation on the anode charge/discharge cycled for 200 cycles, reproducible results were obtained (Figure S17, Supporting Information). This outcome aligns with a prior study conducted by our group.^[^ ^8^ ^]^ The higher resistance observed during the lithiation of the anode, as evidenced by Δ*V* (Figure 1c) analysis, suggests that the SEI formed with 1,8‐NS is likely to be more substantial and resistant compared to the SEI without 1,8‐NS, particularly on the SiO*_x_* particles. This is supported by the d*Q*/d*V* (Figure S4, Supporting Information), indicating a higher overvoltage increase for the lithiation of SiO*_x_* (+70 mV) compared to graphite (+50 mV). It is assumed that this increase is attributable to the degradation products of 1,8‐NS incorporated in the SEI, as monitored with *operando* ATR‐FTIR. These degradation products may increase the stability of the SEI, thereby reducing the prevalence of parasitic reactions. This hypothesis is substantiated by the higher CEs and specific discharge capacities during ongoing charge/discharge cycling in the case of cells with BE+1,8‐NS (Figure 1a,b) compared to cells with BE. Overall, these results indicate the formation of a thicker SEI on the SiO*_x_* particles compared to the graphite particles.^[^ ^8^, ^71^ ^]^ Consequently, this might be due to the continuous SEI (re‐)formation during SiO*_x_* particle volume changes upon (de‐)lithiation. Additionally, a selective involvement of 1,8‐NS in SEI formation on the SiO*_x_* particles is suggested, as the initial lithiation of SiO*_x_* occurs before the initial lithiation of graphite (Figure S4, Supporting Information) and the reduction of 1,8‐NS occurs before the reduction of EC and EMC (Figure 2a).

A notable observation is the much higher intensity of nickel on the SiO*_x_* particle ((220 ± 120) cps) compared to the graphite particle ((40 ± 30) cps). Given the higher specific gravimetric and volumetric capacity of SiO*_x_* compared to graphite, it is anticipated that a greater lithium ion flux density is occurring at the SiO*_x_* particle.^[^ ^11^ ^]^ Consequently, the hypothesis is put forward that the accumulation of Ni on the SiO*_x_* particle is attributable to the overall higher ion flux density at the SiO*_x_* compared to the graphite.

## 3 Conclusion and Outlook

This study investigates the beneficial impact of an optimized concentration of 1,8‐naphthosultone (1,8‐NS) as an electrolyte additive in combination with LiPF_6_ and LiDFP in EC/EMC on the electrochemical performance of high‐voltage (up to 4.5 V) NMC811 || AG+20 % SiO*_x_* multilayer pouch cells is presented and discussed.

**Figure 6:**
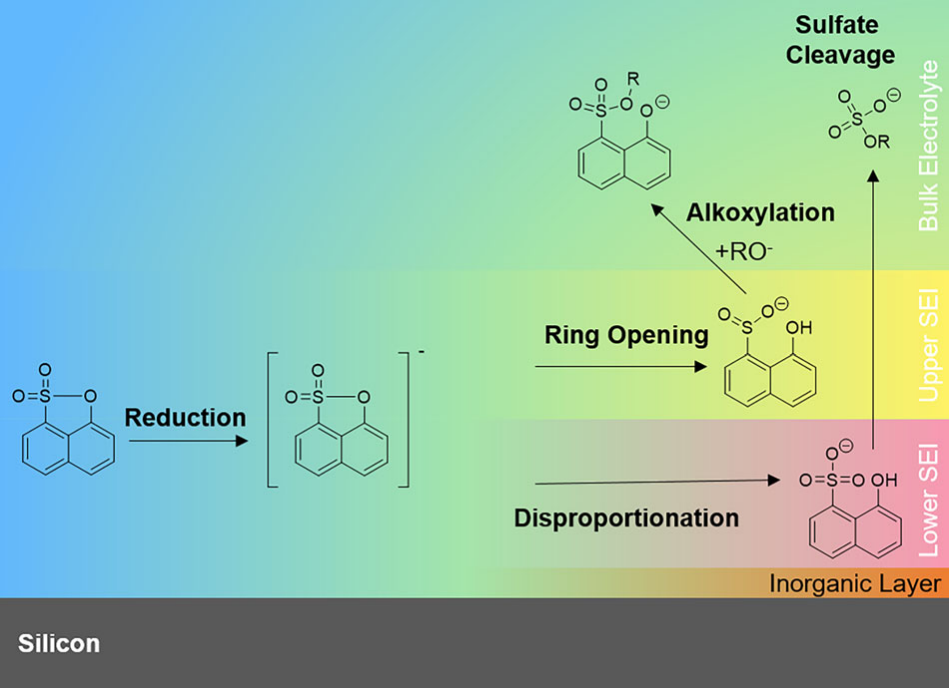
Schematic depiction of the degradation mechanism of 1,8‐NS on the anode surface and the formation of a layered SEI.

The incorporation of 1,8‐NS into the electrolyte formulation has been shown to prolong the cycle life and improve the Coulombic efficiencies of the cells. The combination of 1,8‐NS with LiDFP leads to an even longer cycle life, albeit with an increased cell resistance and slightly lower Coulombic efficiencies. Through *ex situ* GC‐MS, IC‐CD‐MS, and DFT calculations, the identification of electrolyte aging products and the proposal of a putative degradation mechanism have been facilitated. The 1,8‐NS has been identified to degrade to S─O ring‐opened 1‐naphthol‐8‐sulfinate and 1‐naphthol‐8‐sulfonate. The reaction of 1,8‐NS and its degradation products with alkoxides from electrolyte solvent degradation has been shown to result in the formation of aromatic organosulfonates and the cleavage of the sulfur group, resulting in the formation of aliphatic organosulfates and 1‐naphtholate. The investigation of the interphase formation with 1,8‐NS has been conducted through *operando* ATR‐FTIR spectroscopy, which has revealed the reduced 1,8‐NS as a relatively stable intermediate product. Furthermore, a two‐layered interphase model, comprising 1‐naphthol‐8‐sulfinate in the upper layer toward the bulk electrolyte and 1‐naphthol‐8‐sulfonate in the lower layer toward the electrode, is proposed (**Figure** **6**). This model suggests an enhancement in the stability and flexibility of the interphase, attributable to the potential for establishing *π–π* interactions with the aromatic backbone, thereby hosting volumetric changes of SiO*_x_* particles. It is further anticipated that the ionic conductivity of the interphase formed with 1,8‐NS will be retained, given the presence of organic sulfinate and sulfonate groups. A *post mortem* investigation with SEM and EDX indicates a thicker interphase on SiO*_x_* particles than on graphite particles and a preferred accumulation of 1,8‐NS degradation products, as notably increased amounts of sulfur, phosphorus, and fluorine are found. Follow‐up studies might address this phenomenon by investigating the differences in composition and structure between the SEIs on SiO*_x_* and graphite in composite electrodes that are formed with different electrolyte additives. Additionally, follow‐up studies might address the formation mechanism of aliphatic organosulfates from the 1,8‐NS degradation products.

## 4 Experimental Section

### Electrolyte Preparation and Other Chemicals

The baseline electrolyte (BE) utilized was 1.00 m LiPF_6_ (E‐Lyte Innovations GmbH, Germany) in EC/EMC (3:7, by weight; E‐Lyte Innovations GmbH, Germany). The electrolyte additives lithium difluorophosphate (LiDFP, TCI Chemicals GmbH, Germany) and 1,8‐Naphthosultone (1,8‐NS, abcr GmbH, Germany) were incorporated into the electrolyte solution. The electrolytes were prepared and stored in an argon‐filled glovebox (O_2_ and H_2_O < 5 ppm, MBraun, Germany). For further experiments, DMC (E‐Lyte Innovations GmbH, Germany), LiBF_4_ (E‐Lyte Innovations GmbH, Germany), sodium 1‐naphthol‐8‐sulfonate (TCI), lithium methoxide (TCI), 1‐naphthol (TCI), and sodium 1‐naphthalenesulfonate (TCI) were used as received.

### Electrochemical Investigation

For electrochemical investigations, multilayer wounded NMC811 || AG+20 % SiO*_x_* pouch cells, balanced for cell operation ranging up to 4.5 V, were obtained from LiFUN Technology Corporation Limited (Hunan, China) in a sealed and dry state. The cathode exhibits an active mass loading of 15.28 mg cm^−2^ with an overall active material fraction of 96.4 % and the anode exhibits an active mass loading of 6.45 mg cm^−2^ with an overall active material fraction of 94.5 %. The electrode areas were 88.14 cm^2^ and 99.4 cm^2^, resulting in an N/P ratio of 1.4. The electrodes were double‐side coated on 12 µm aluminum and 8 µm copper current collectors. The cells were opened and dried overnight at 90 °C under reduced pressure before cell assembly. Then the pouch cells were filled with 700 µL electrolyte (corresponding to 4.2 g Ah^−1^ for BE and 200 mAh nominal capacity at 0.1C of the used pouch cells). Subsequently, the pouch cells were vacuum sealed at 15 % of the ambient pressure (GN‐HS350V, Gelon Lib Co., Ltd., China), while maintaining a gas pocket, in a dry‐room (dew point: < −50 °C). Following two CCCV charge and CC discharge formation cycles at 0.1C, the cells were cut open, vacuum‐sealed directly at the cell stack (“degassing”), and then charge/discharge cycled at 1C (200 mA) until 50 % SoH. The cells were charged to an upper cell voltage cut‐off of 4.5 V in a constant‐current constant‐voltage (CCCV) procedure until the current dropped below 0.01C and CC discharged to a lower cell voltage cut‐off of 2.8 V. Maccor 4000 battery testers with 20 °C temperature chambers were used for the galvanostatic charge/discharge investigations. During the operation, the cells were clamped with a custom‐built cell holder, as previously reported in,^[^ ^72^ ^]^ to apply a stack pressure of ≈2 bar. Four cells per electrolyte variation were investigated. For the calculation of the SoH, the fourth overall cycle (second cycle at 1C) was used as a reference point for maximum capacity.

### Theoretical Calculations

Density functional theory (DFT) calculations were carried out using the Gaussian16 package.^[^ ^73^ ^]^ All geometries were optimized using the B3LYP DFT functional and the 6‐311++G(3df,2p) basis set. The effect of a surrounding electrolyte was mimicked by SMD implicit solvation model using parameters for acetone, showing a similar dielectric constant as liquid carbonate‐based electrolytes.^[^ ^7^, ^8^, ^74^, ^75^, ^76^ ^]^ The electronic energies for the putative reactions were calculated with thermal energy correction (298.15 K). For the calculations, the “xQC” SCF convergence criterion and ultrafine integration grid were used. To relate the value of the calculated reduction potential to the Li|Li^+^ scale, a shift of −1.4 V was applied. The bond orders were obtained by natural bond orbital (NBO) analysis, using the “pop = nboread” and the “$nbo bndidx $end” keywords within the DFT calculation. The predicted infrared spectra were calculated using the “freq” keyword, and the calculated wavenumbers in the predicted infrared spectra were reported as was (*x* = 1.00), and with *x* = 0.98 as scaling factors.

### Ion Chromatography‐Conductivity Detection‐Mass Spectrometry

A qualitative analysis of anionic species in the extracted electrolyte was executed on an 850 Professional IC (Metrohm, Switzerland) with conductivity detection (CD) hyphenated to a 6530 Accurate Mass Quadrupole‐Time‐of‐Flight (Q‐TOF)‐MS (Agilent, USA). For an isocratic separation of the anionic compounds, a Metrosep A Supp 7 column (250 x 4.0 mm, 5 µm; Metrohm) with a Metrosep A Supp 5 Guard/4.0 guard column at an oven temperature of 65 °C and an applied flow rate of 0.7 mL min^−1^ over a total runtime of 30 min was used. All samples were diluted 1:100 with acetonitrile and the injection volume was set to 65 µL. The eluent consisted of a 3.6/3.4 mm Na_2_CO_3_/NaHCO_3_ aqueous solution and acetonitrile in a ratio of 58:42 (v/v). The utilized suppressor was sequentially regenerated by 0.1 m sulfuric acid and rinsed with MilliQ water. Ionization in the MS was performed in ESI(‐) mode at a capillary voltage of 3.5 kV. The nebulizer gas was set to 45 psig and drying gas to a flow of 10 L min^−1^ at 350 °C. A collision‐induced dissociation energy of 30 eV was applied for MS/MS experiments. The mass range was set to *m*/*z* 70‐500 in MS^1^ and *m*/*z* 50‐500 in MS^2^. Instrument control, data acquisition, and data evaluation were performed for MS with MassHunter Data Acquisition and MassHunter Qualitative Analysis B.08.00 (Agilent), while for IC with MagIC Net 3.3 (Metrohm).

To obtain electrochemically aged electrolytes, the pouch cells were galvanostatically charged to the cut‐off voltages 2.6, 3.3, and 4.5 V and were opened in a glovebox (MBraun, Germany, O_2_, H_2_O contents <0.1 ppm). The electrolytes were extracted *via* centrifugation of the anode and separator and transferred to GC vials in a dry‐room.

### Gas Chromatography‐Mass Spectrometry

A qualitative analysis of volatile electrolyte species was executed on a Nexis GC‐2030 (Shimadzu) equipped with a non‐polar Restek Rxi‐5ms (30 m x 0.25 mm x 0.25 µm) fused silica column (5 % diphenyl/ 95 % dimethyl polysiloxane). The sample injection was done with a volume of 1 µL at an applied split ratio of 1:10 and a set temperature of 250 °C. Helium (6.0 purity) was used as carrier gas with a column flow of 1.15 mL min^−1^. The oven program started with an initial temperature of 40 °C which was held for 1 min, followed by a first ramp of 3 °C min^−1^ °C to 60 °C and a subsequent second ramp of 30 °C min^−1^ until a temperature of 260 °C was reached. This final temperature was held for 2 min, resulting in a total measurement time of 16.33 min. The MS operated in the electron ionization (EI) mode with an ion source temperature of 200 °C and an interface temperature of 250 °C. The filament voltage was set to 70 V and the detector voltage was relative to the respective tuning file. The mass range was set to 30‐350 *m*/*z* with an event time of 0.1 s in scan mode. The identification of 1,8‐Naphthosultone was verified with the NIST 11 library. All samples were diluted 1:100 with DCM to precipitate and remove the conductive salt prior to injection.

### Operando Attenuated Total Reflection Fourier‐Transform Infrared Spectroscopy

*Operando* ATR‐FTIR measurements were carried out on an Invenio‐R (Bruker, US) with a mercury‐cadmium‐telluride (MCT) detector on a ZnSe ATR crystal (Bruker, US) and a VeeMAX III automatic variable angle specular reflection accessory (PikeTechnologies, US) in a self‐built spectroelectrochemical cell. For detailed information about the cells, the reader was referred to a previous study.^[^ ^8^ ^]^ In this study, an additional lithium metal reference and a second glass fiber separator (Whatman GF/D, 13 mm diameter) were added. The lithium metal reference was contacted by a small stainless‐steel foil covered with a polyimide foil to prevent short circuits. The separators were wetted with 150 µL electrolyte each. The spectral resolution was 4 cm^−1^ and each spectrum was obtained by accumulating 32 sample scans and 32 background scans. A second cell without electrolyte was inserted into the spectrometer for background scans. All spectra were processed with H_2_O correction. Additionally, concave rubberband background correction was carried out with 33 iterations to increase comparability at different incident angles. Electrochemical tests were performed with an Autolab PGSTAT204 (Metrohm, Germany) controlled by the NOVA 2.1 software. The cell was polarized from open circuit voltage (OCV) to 0.005 V with a scan rate of 0.4 mV s^−1^. The IR measurements were carried out during cell operation. Note that there was a native oxide layer on the silicon wafer surface, resembling the SiO*_x_* particles present in the AG + 20 % SiO*_x_* anode. Spectra of bulk electrolytes and additives were recorded with a Platinum ATR accessory with a diamond crystal (Bruker, US). The IR spectrometer was located in a box with a nitrogen atmosphere which was constantly flushed with nitrogen.

### Scanning Electron Microscopy and Energy‐Dispersive X‐ray Spectroscopy Analysis

Further SEM images were recorded with a Zeiss Crossbeam 550 electron microscope (Carl Zeiss Microscopy GmbH). A 3 kV accelerating voltage, an aperture size of 30 µm, and an in‐lens detector with a working distance of ≈ 5 mm were used. To not induce any surface changes by the electron beam during exposure at high magnifications, the acquisition time was optimized. The investigated electrochemically aged electrodes were taken from NMC811 || AG+20 % SiO*_x_* pouch cells charge/discharge cycled with BE+1,8‐NS (as described above). Prior to SEM and EDX measurements, the electrodes were rinsed with 100 µL DMC in an argon‐filled glovebox (O_2_, H_2_O contents <0.1 ppm), dried under reduced pressure, and transferred in an air‐tight sample chamber to prevent air exposure.

Energy dispersive X‐ray spectroscopy with an acceleration voltage of 5 kV was measured with an Ultim Extreme detector to evaluate the elemental composition of the samples. The spectra were evaluated with the Integrated Calibration and Application Tool (INCA) software (Oxford Instruments).

## Conflict of Interest

The authors declare no conflict of interest.

## Supporting information



Supporting Information

## Data Availability

The data that support the findings of this study are available from the corresponding author upon reasonable request.
