# Supplementary material for: Drawing from the Old‐The First Ever Sultone as Electrolyte Additive in High‐Voltage NMC811 || AG+SiOx Multilayer Pouch Cells
Source: Small. 2025 Aug 25;21(41):e07089. doi: 10.1002/smll.202507089 (PMC12530034; doi:10.1002/smll.202507089)
Supplement: Supplementary file 1 — Supporting Information [file SMLL-21-e07089-s001.docx]

# **Supporting Information**


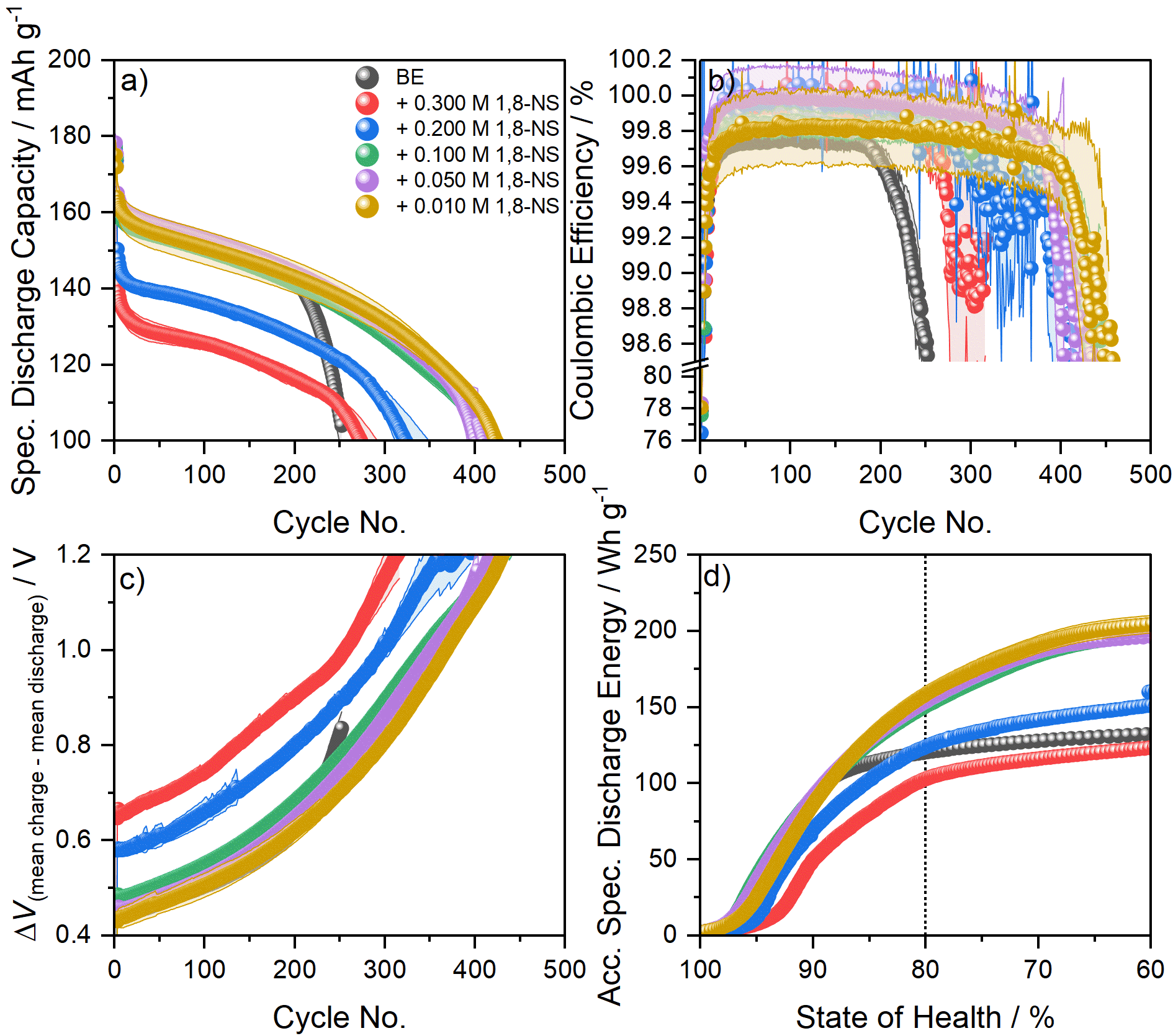


Figure S1: a) Specific discharge capacities, b) Coulombic efficiencies, c) ΔV vs. cycle number, and d) accumulated specific discharge energy vs. state-of-health of NMC811‖AG+20 % SiO_x_ multilayer pouch cells with 1.00 m LiPF_6_ in EC/EMC (3:7, by weight) as baseline electrolyte (BE) and with different concentrations of 1,8-Naphthosultone (1,8-NS).

A comparison with other commonly used electrolyte additives 1,3,2-dioxathiolane 2,2-dioxide (DTD), fluoroethylene carbonate (FEC), and vinylene carbonate (VC) is shown in Figure S3 and Table S1.

In a previous study on the electrolyte additive 2-SBA, similar NMC811‖AG+20 % SiO*_x_* pouch cells were used.^[8]^ However, the cells with BE in that study failed already after 65 cycles, whereas in this study the cells with BE failed after (195 ± 5) cycles. This might be due to two main reasons, which are that a current of 0.20 A (=1C) is used here instead of a current of 0.25 A (=1C) in the previous study, with the higher current expected to lead to a faster cell failure, such as rollover, and second the use of a new batch of the purchased cells. Still, a fair comparison of 2-SBA and 1,8-NS in the same cells with the same current (0.25 A) is shown in Figure S3 and Table S1, exhibiting a longer cycle life of the cells with 1,8-NS compared to 2-SBA.


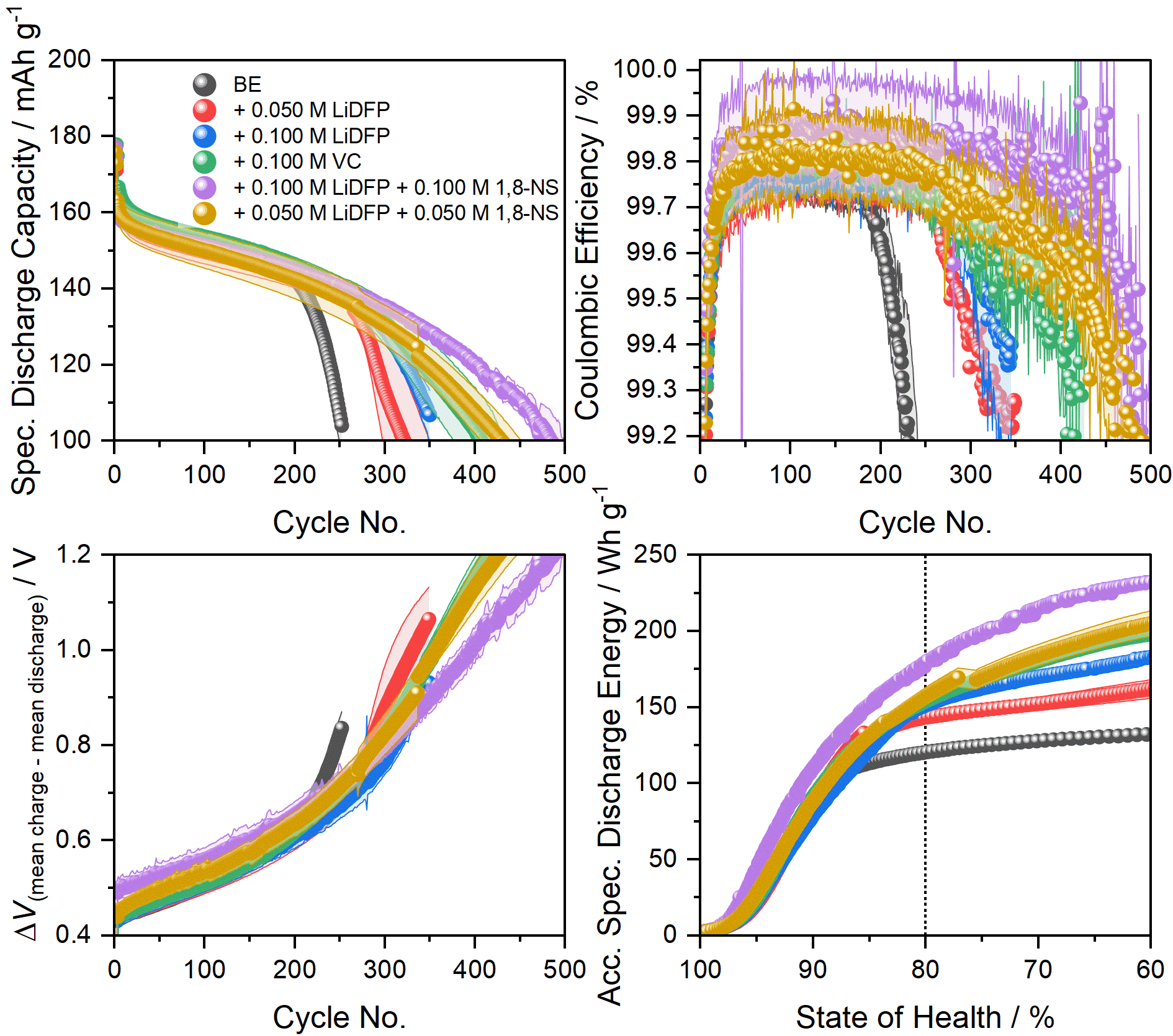


Figure S2 a) Specific discharge capacities, b) Coulombic efficiencies, c) ΔV vs. cycle number, and d) accumulated specific discharge energy vs. state-of-health of NMC811‖AG+20 % SiO_x_ multilayer pouch cells with 1.00 M LiPF_6_ in EC/EMC (3:7, by weight) as baseline electrolyte (BE) and with different concentrations of lithium difluorophosphate (LiDFP), mixtures LiDFP with 1,8-Naphthosultone (1,8-NS), mixture of 0.1 m LiDFP with 0.1 m vinylene carbonate (VC), and 0.1 m VC.


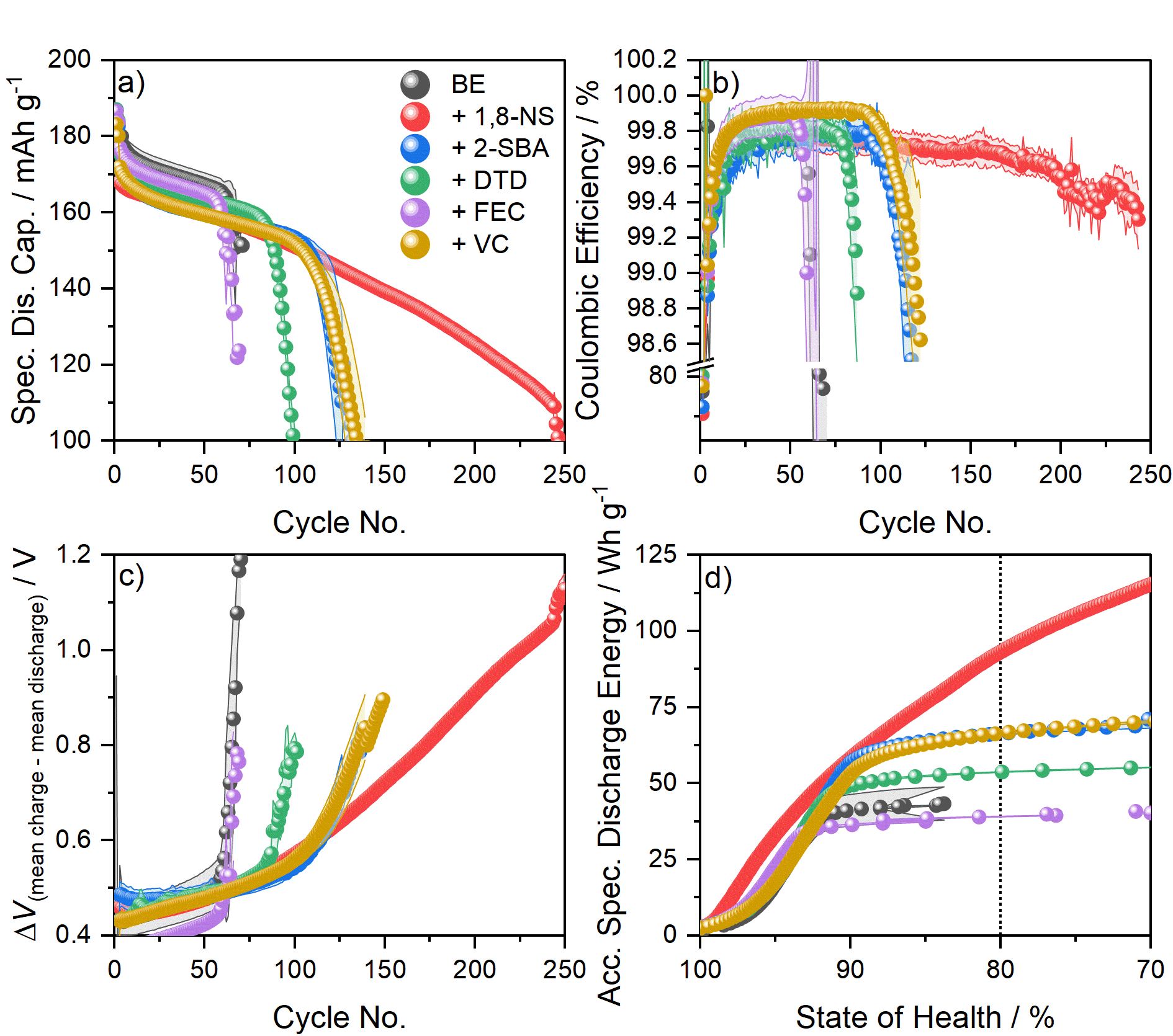


Figure S3: a) Specific discharge capacities, b) Coulombic efficiencies, c) ΔV vs. cycle number, and d) accumulated specific discharge energy vs. state-of-health of NMC811‖AG+20 % SiO_x_ multilayer pouch cells with 1.00 m LiPF_6_ in EC/EMC (3:7, by weight) as baseline electrolyte (BE) and with 0.15 m 1,8-NS, 0.10 m 2-SBA, 0.10 m of 1,3,2-dioxathiolane 2,2-dioxide (DTD), 0.15 m fluoroethylene carbonate (FEC), 0.15 m vinylene carbonate (VC), and. Experimental details and further analysis can be found in ref ^[8]^. Note, that in this Figure 1C equals 250 mA instead of 200 mA in the rest of this manuscript.

Table S1: Cycles until 80 % State-of-Health (SoH), cycles until cell failure, accumulated specific discharge energy until 80 % SoH, Coulombic efficiencies (CE) during ongoing galvanostatic cycling, and initial Δ*V* (mean charge voltage – mean discharge voltage) at 1C of the cells shown in Figure S1, Figure S2, and Figure S3.

| Electrolyte variation | Cycles  until  80 % SoH | Cycles  until  cell failure | Acc. spec. disch. energy until 80 % SoH / Wh g^‑1^ | CE during ongoing galv. cycling / % | Initial Δ*V* at 1C / mV |
| --- | --- | --- | --- | --- | --- |
| BE | 226 ± 1 | 195 ± 5 | 120 ± 1 | 99.75 ± 0.05 | 440 ± 20 |
| + 0.300 m  1,8-NS | 236 ± 4 **+4 %** | 240 ± 3 **+23 %** | 103 ± X **-14 %** | 99.9 ± 0.1 | 648 ± 5 |
| + 0.200 m  1,8-NS | 263 ± 4 **+16 %** | 240 ± 10 **+23 %** | 124 ± 1 **+3 %** | 99.94 ± 0.05 | 580 ± 20 |
| + 0.100 m  1,8-NS | 290 ± 5 **+31 %** | 385 ± 5 **+97 %** | 150 ± 2 **+25 %** | 99.85 ± 0.1 | 486 ± 3 |
| + 0.050 m  1,8-NS | 295 ± 4 **+31 %** | 385 ± 5 **+97 %** | 156 ± 2 **+30 %** | 100.0 ± 0.2 | 460 ± 20 |
| + 0.010 m  1,8-NS | 302 ± 5 **+34 %** | 405 ± 5 **+108 %** | 156 ± 2 **+30 %** | 99.80 ± 0.02 | 430 ± 20 |
| + 0.05 M LiDFP +0.05 M 1,8-NS | 302 ± 6 **+34 %** | 430 ± 10 **+121 %** | 157 ± 2 **+31 %** | 99.8 ± 0.1 | 441 ± 8 |
| + 0.1 M LiDFP +0.1 M 1,8-NS | 351 ± 5 **+55 %** | 460 ± 5 **+136 %** | 180 ± 2 **+50 %** | 99.9 ± 0.1 | 500 ± 20 |
| + 0.1 M VC | 290 ± 5 **+28 %** | 275 ± 25 **+41 %** | 152 ± 2 **+26 %** | 99.80 ± 0.02 | 440 ± 10 |
| + 0.1 M LiDFP | 287 ± 3 **+26 %** | 280 ± 5 **+44 %** | 150 ± 3 **+25 %** | 99.78 ± 0.05 | 430 ± 20 |
| + 0.05 M LiDFP | 275 ± 1 **+22 %** | 265 ± 5 **+36 %** | 143 ± 1 **+19 %** | 99.78 ± 0.05 | 430 ± 20 |
|  |  |  |  |  |  |
| Old batch BE^[8]^ | 70 ± 1 | 60 ± 1 | 42 ± 1 | 99.79 ± 0.09 | 470 ± 80 |
| + 0.15 m 1,8-NS | 170 ± 3 **+143 %** | 244 ± 4 **+301 %** | 92 ± 1 **+119 %** | 99.7 ± 0.1 | 460 ± 5 |
| + 0.10 m 2-SBA | 117 ± 1 **+67 %** | 95 ± 3 **+58 %** | 67 ± 1 **+160 %** | 99.8 ± 0.1 | 490 ± 30 |
| + 0.10 m DTD | 92 ± 1 **+31 %** | 80 ± 2 **+33 %** | 54 ± 1 **+29 %** | 99.8 ± 0.1 | 433 ± 9 |
| + 0.15 m FEC | 65 ± 1 **-7 %** | 50 ± 2 **-17 %** | 39 ± 1 **-7 %** | 99.9 ± 0.1 | 370 ± 5 |
| + 0.15 m VC | 118 ± 1 **+69 %** | 95 ± 3 **+58 %** | 66 ± 1 **+57 %** | 99.91 ± 0.03 | 430 ± 20 |


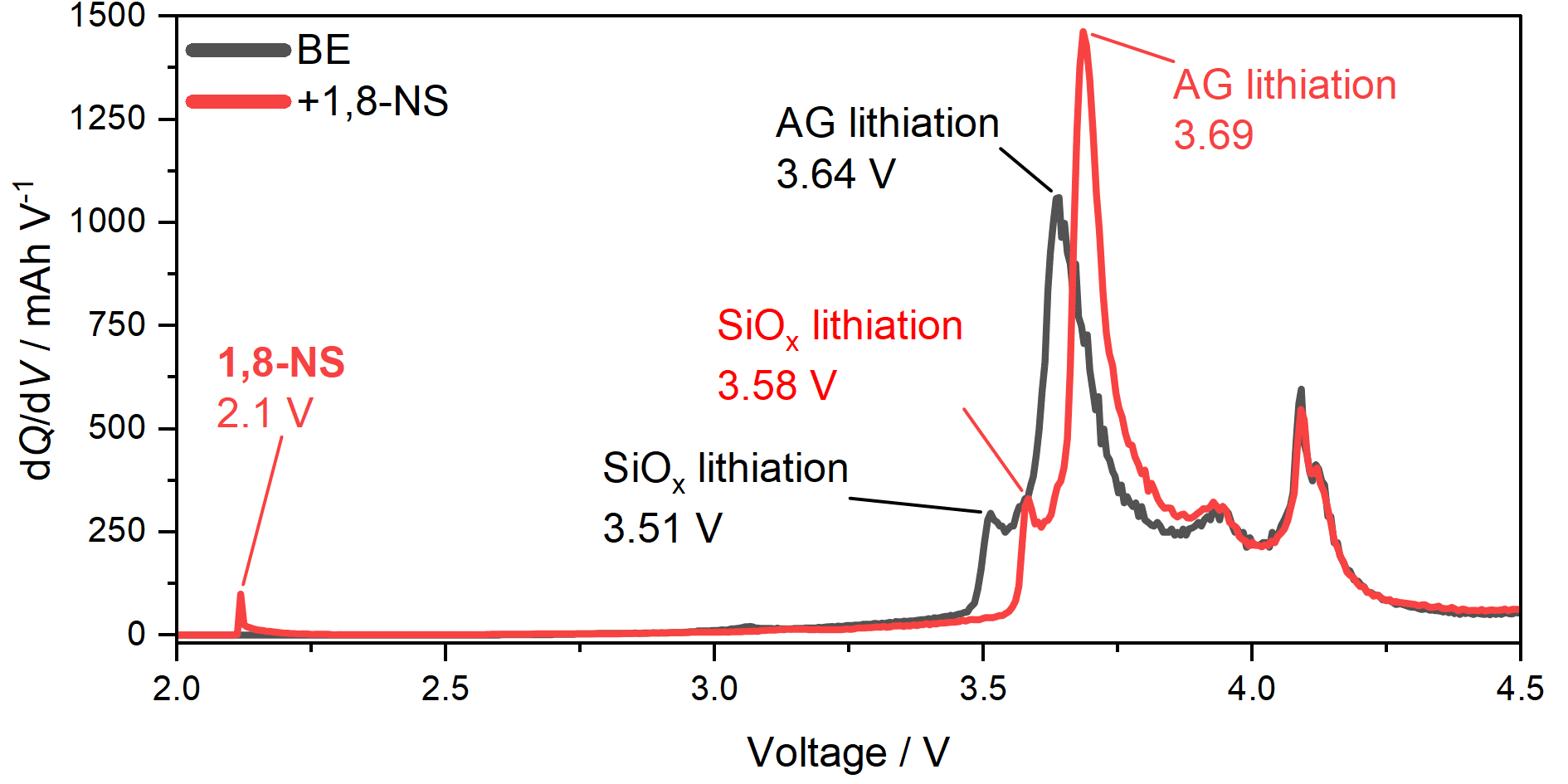


Figure S4: dQ/dV voltammogram of the first charge up to 4.5 V of NMC811‖AG+20 % SiO_x_ multilayer pouch cells with BE (1 m LiPF_6_ in EC/EMC (3:7 by wt.%)) and BE + 0.1 m 1,8-Naphthosultone (1,8-NS).

Figure S5: Putative reduction of 1,8-NS and subsequent C-O, S-O, or C-S ring opening products of reduced 1,8-NS with the calculated ΔE and ΔG.


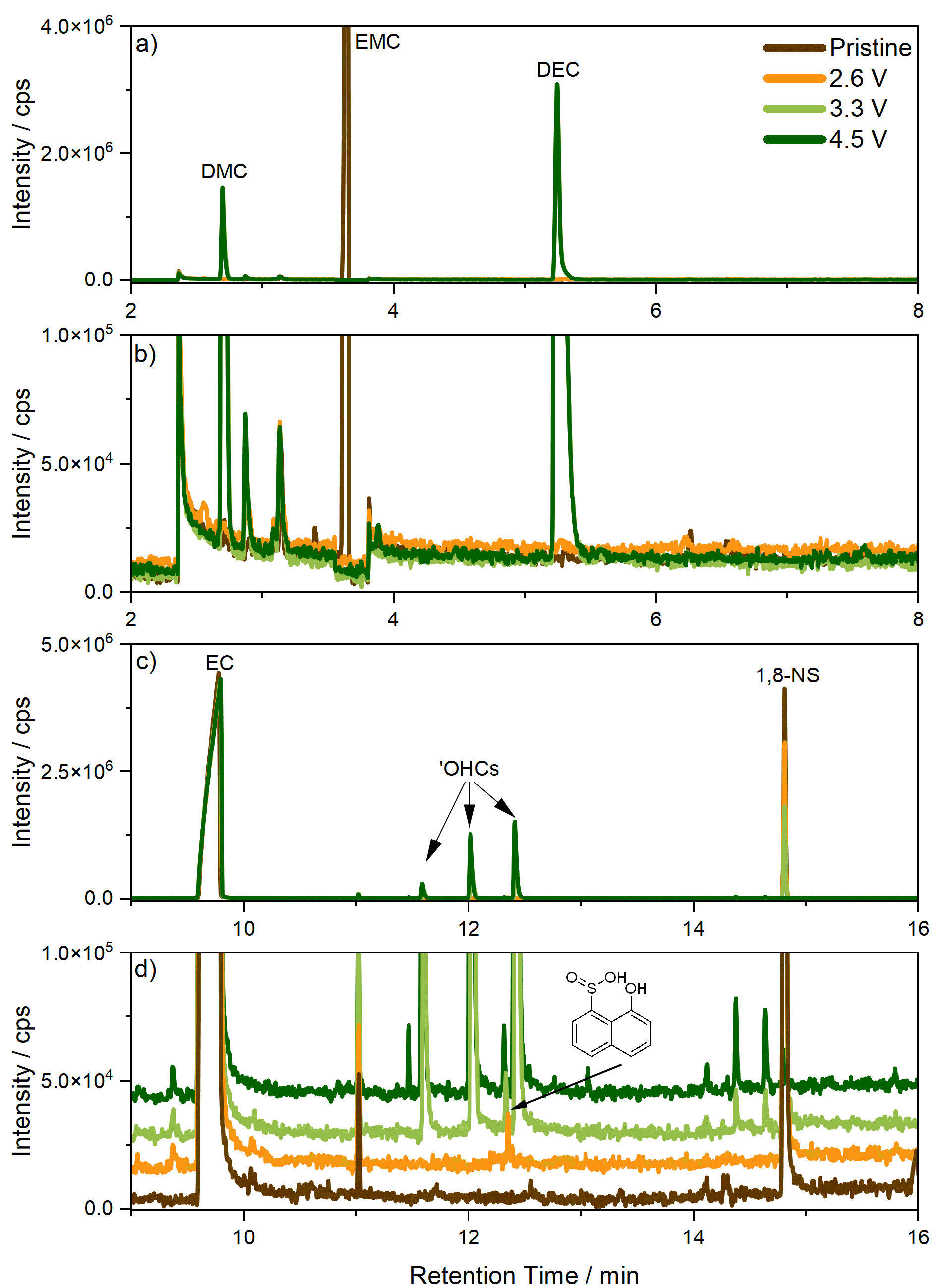


Figure S6: a, c) GC-MS chromatogram of pristine electrolyte and electrolyte samples extracted from NMC811‖AG+20 % SiO_x_ multilayer pouch cells with BE (1 m LiPF_6_ in EC/EMC (3:7 by wt.%)) + 0.1 m 1,8-Naphthosultone (1,8-NS) charged up to 2.6 V, 3.3 V, and 4.5 V. b) section of a); d) section of c) with shifted baselines for better visibility.


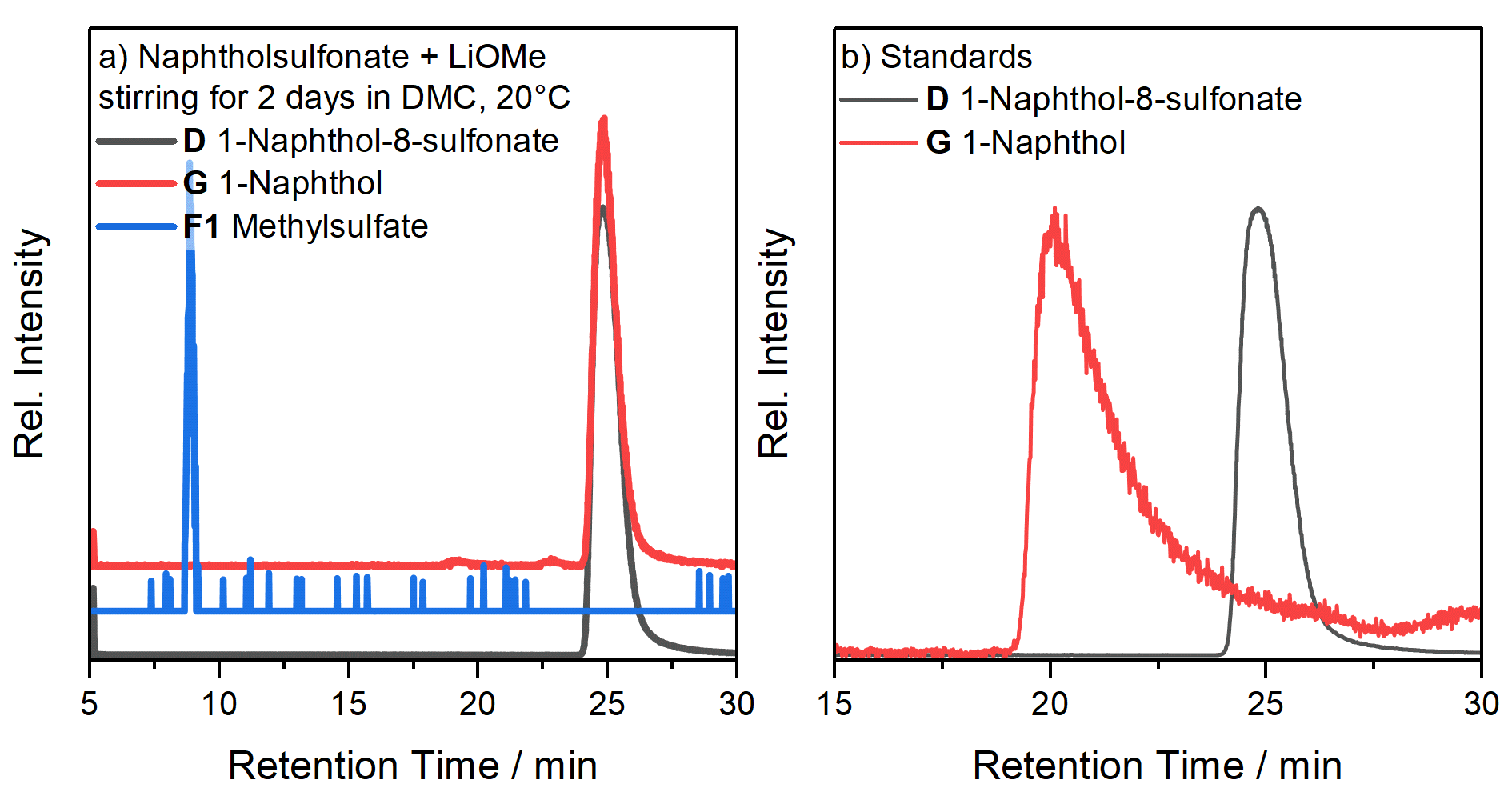


Figure S7: a) EICs of 1-Naphthol-8-sulfonate, methylsulfate, and 1-Naphthol from a solution containing 1-Naphthol-8-sulfonate and LiOMe after stirring for 2 days at 20°C. b) EICs of 1-Naphthol-8-sulfonate (24.81 min) and 1-Naphthol (20.01 min).


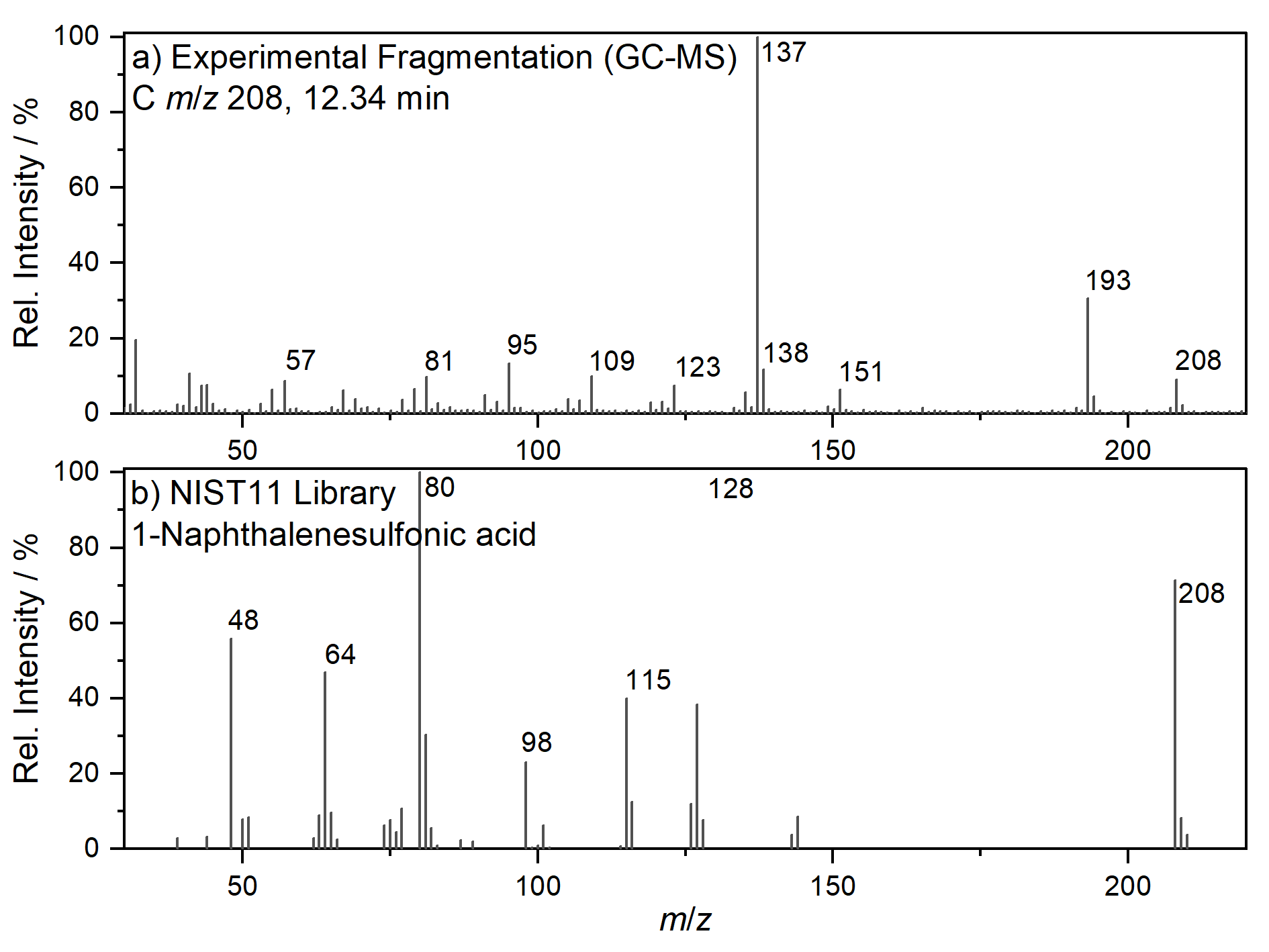


Figure S8: Fragmentation patterns of a) m/z 208 at 12.34 min found in the GC-MS investigation, and b) 1-Naphthalenesulfonic acid from the NIST11 Library


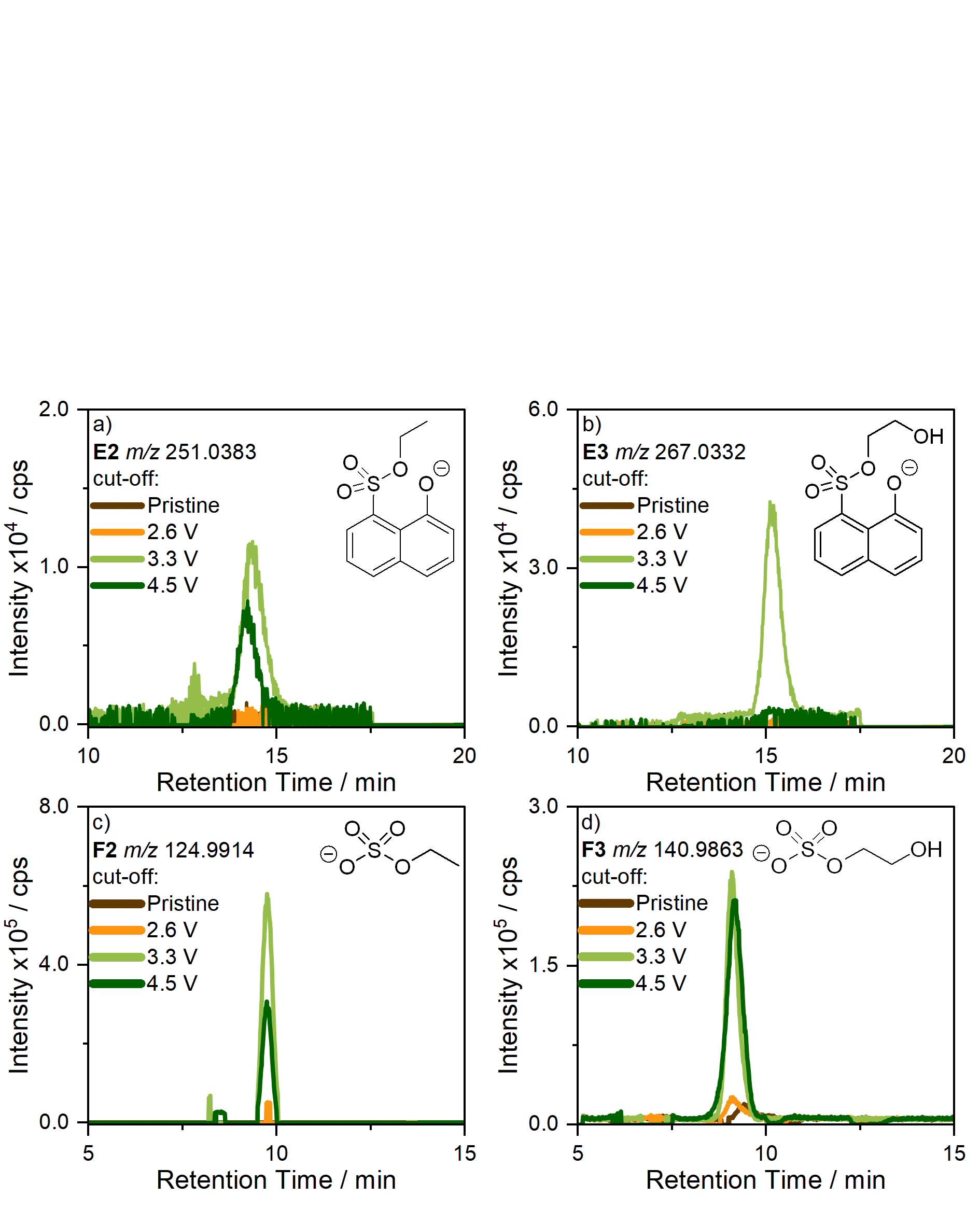


Figure S9: IC-CD-MS EIC chromatogram of **E2**, **E3**, **F2**, and **F3**. The electrolyte samples were obtained from the pristine electrolyte and extracted electrolytes from cells charged to 2.6 V, 3.3 V, and 4.5 V.


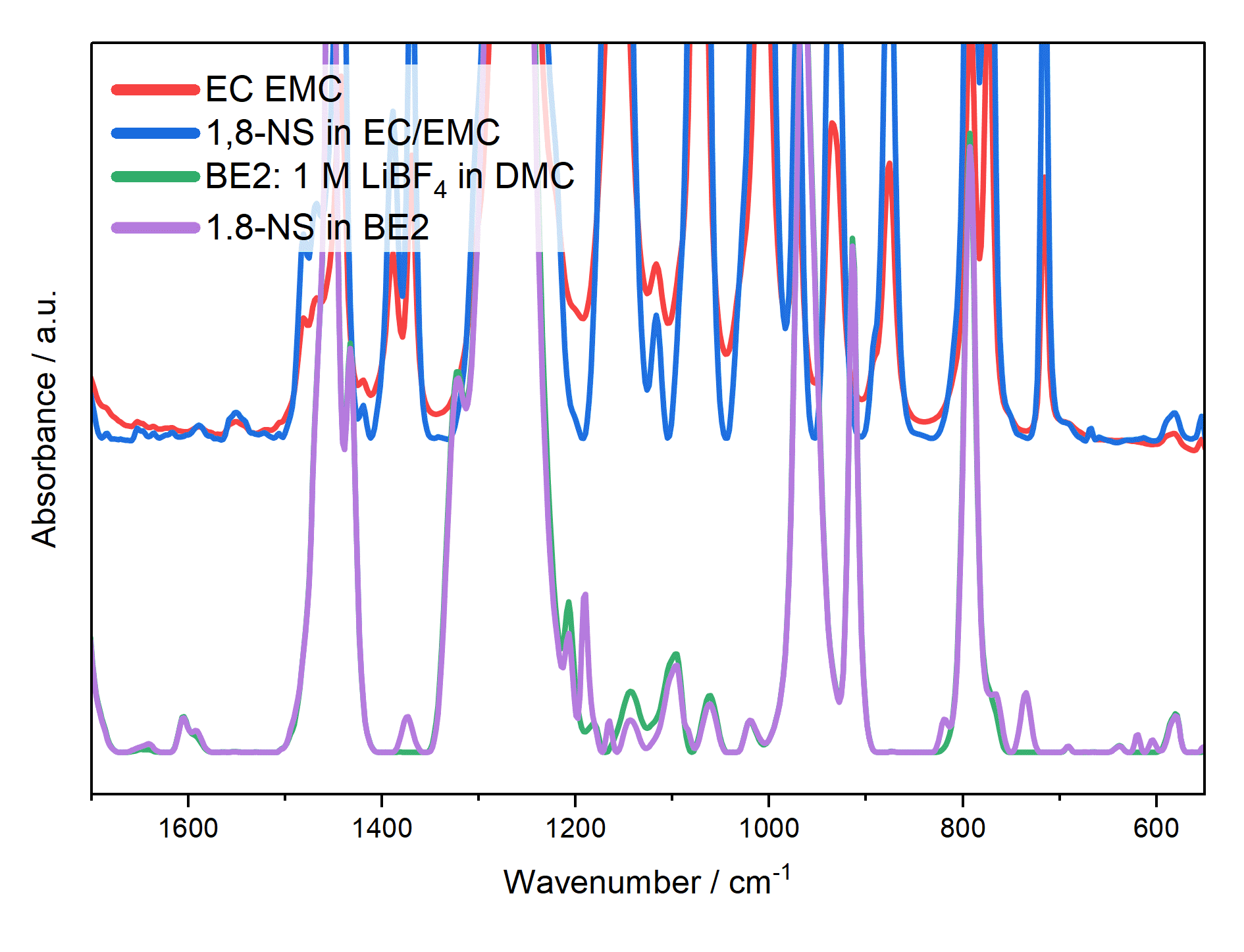


Figure S10: Spectra of EC/EMC (3:7), 1,8-NS in EC/EMC, BE2: 1 m LiBF4 in DMC, and 1,8-NS in BE2.


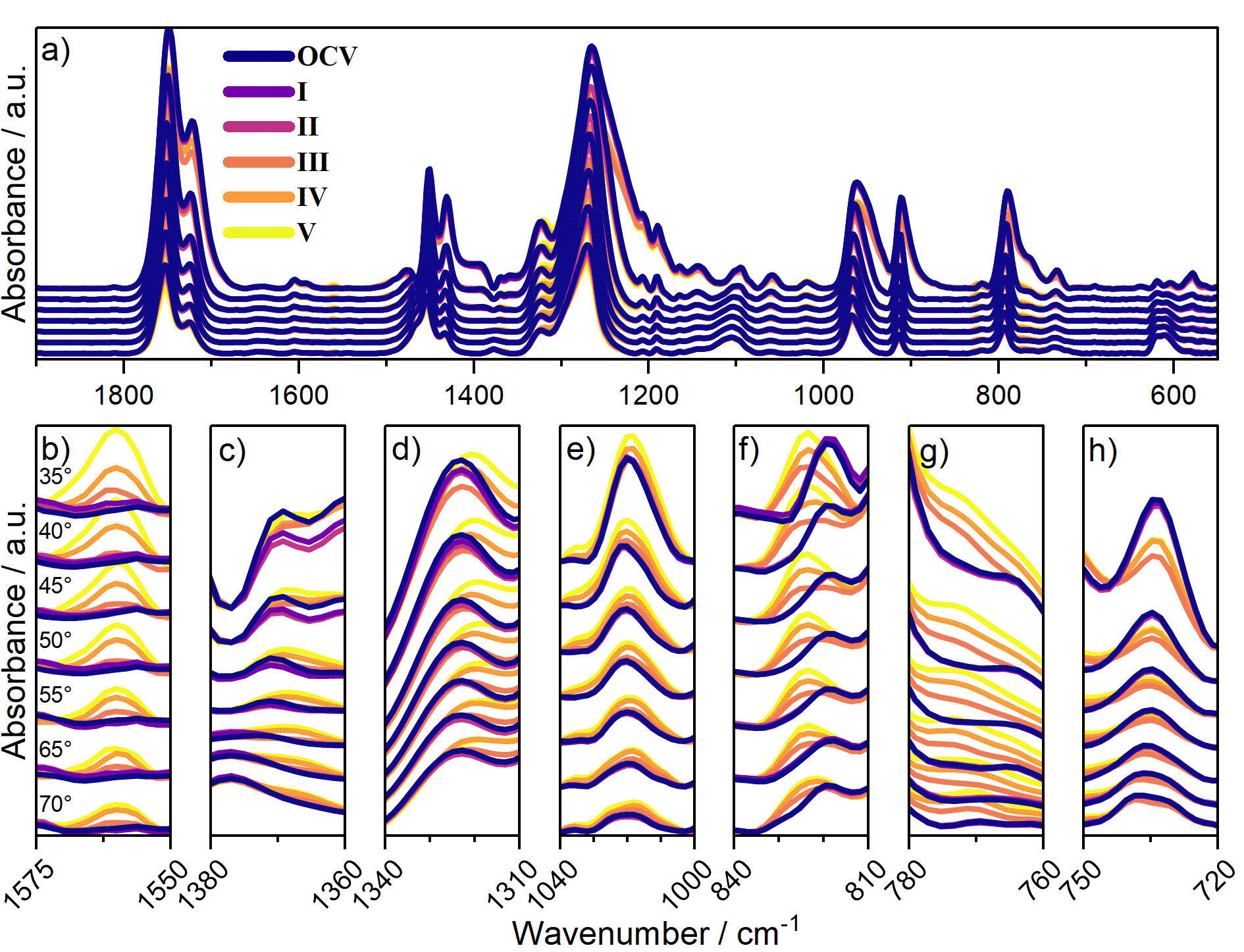


Figure S11: a) ATR-FTIR spectra obtained with 1 m LiBF_4_ in DMC and 1,8-NS in the operando mode with a Si || Li spectroelectrochemical cell with a lithium reference electrode. b-h) sections of the respective regions.


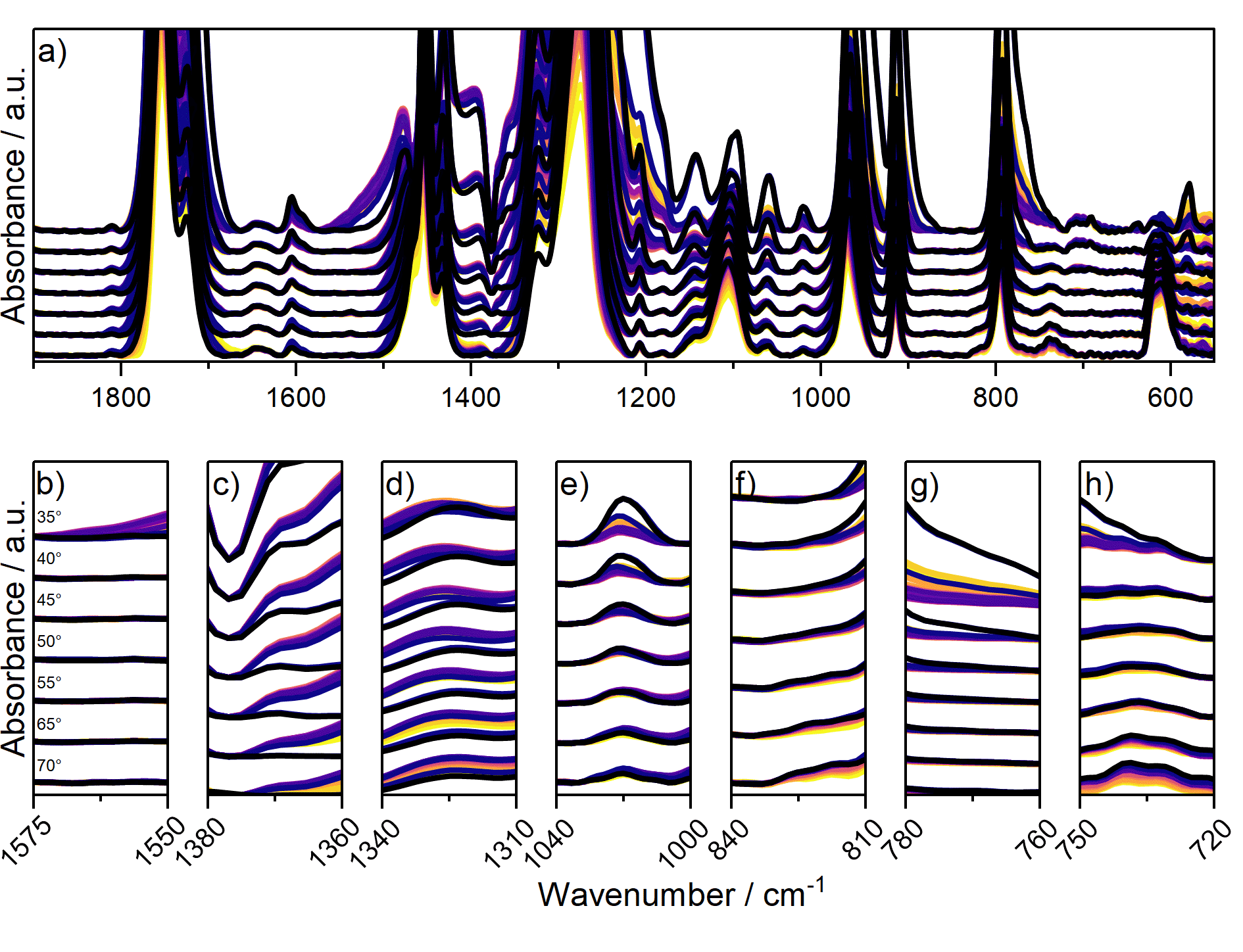


Figure S12: a) ATR-FTIR spectra obtained with 1 m LiBF_4_ in DMC without 1,8-NS in the operando mode with a Si || Li spectroelectrochemical cell with a lithium reference electrode. b-g) sections of the respective regions.


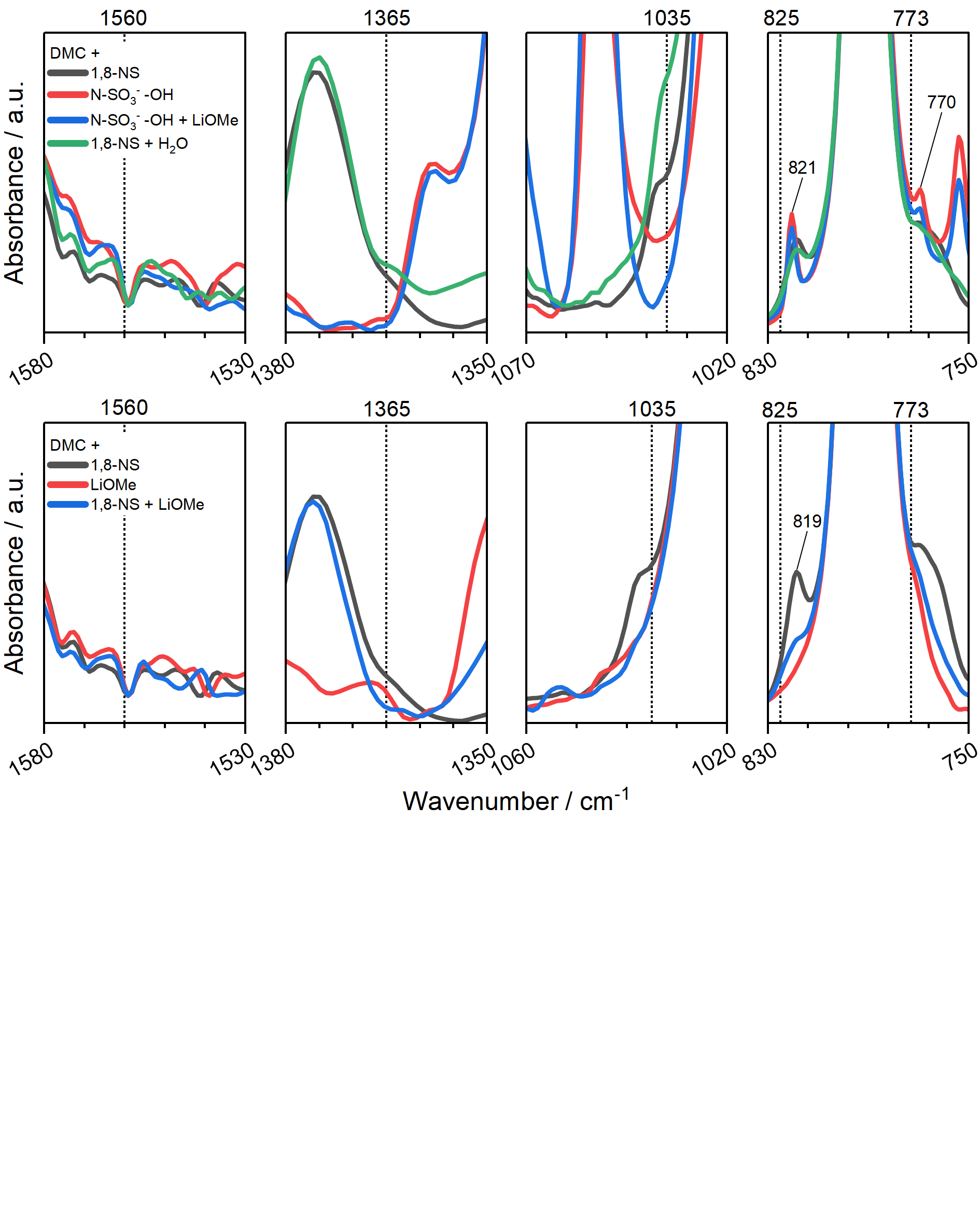


Figure S13: IR spectra of 1,8-NS, 1,8-NS + H_2_O, 1,8-NS + lithium methoxide, sodium 1-naphthol-8-sulfonate, and sodium 1-naphthol-8-sulfonate + lithium methoxide mixtures in DMC.


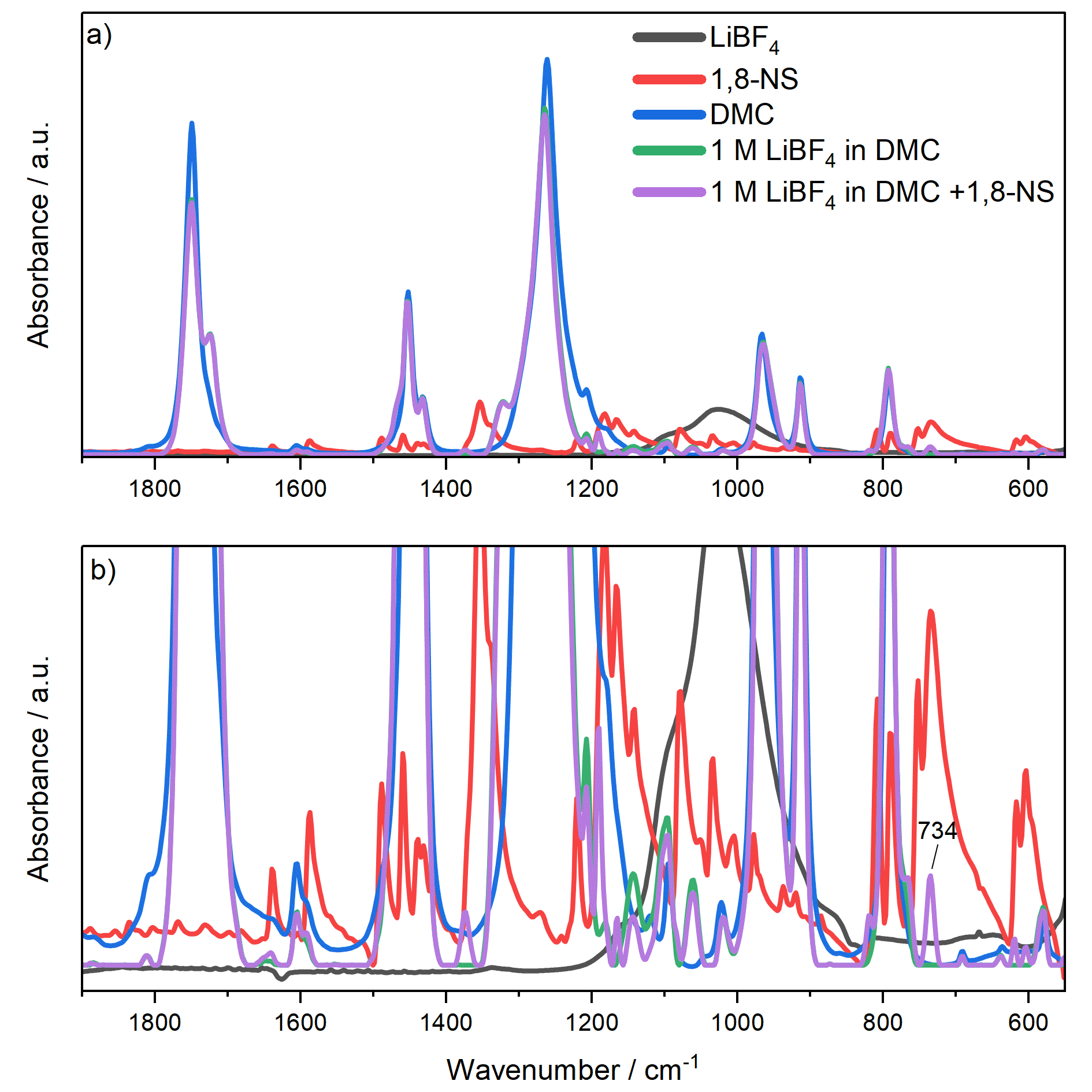


Figure S14: a) Reference spectra of LiBF_4_, 1,8-NS, DMC, 1 m LiBF_4_ in DMC, and 1,8-NS in 1 m LiBF_4_ in DMC. b) Enlarged section of a).


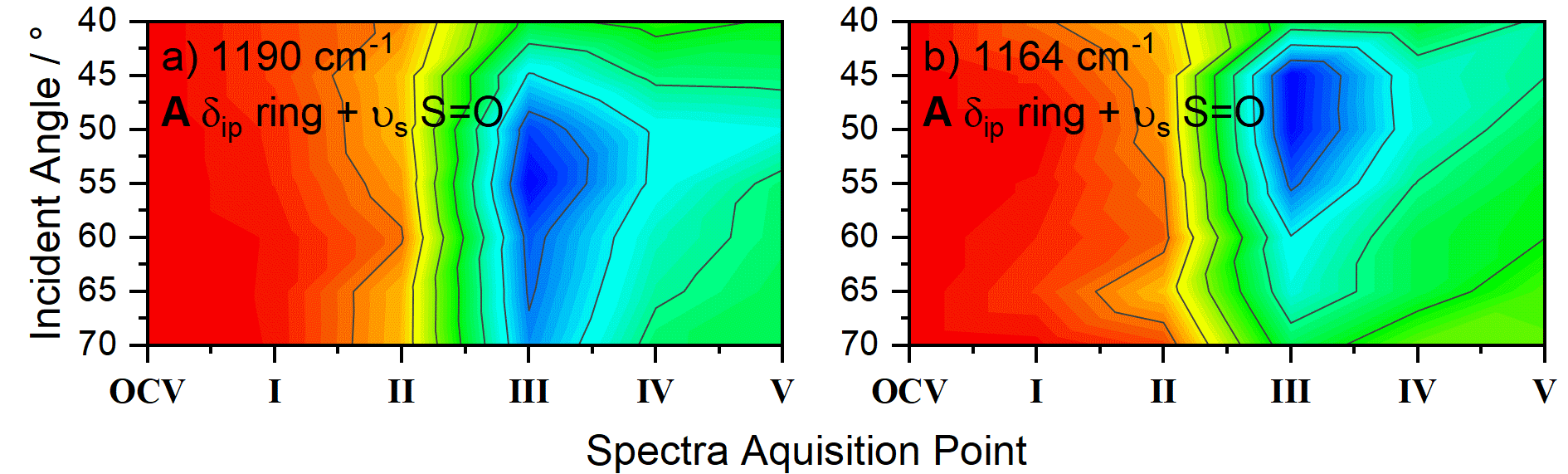


Figure S15: Heatmaps of bands around a) 1190 cm^-1^ and b) 1164 cm^-1^.

Table S2: Experimentally obtained and calculated wavenumbers with a correction factor of x=1.00 and x=0.98 and calculated intensity of infrared bands of 1,8-Naphthosultone. The vibrational modes are assigned by visual evaluation.

| **1,8-Naphthosultone (A)** | | | | |
| --- | --- | --- | --- | --- |
| Experimental  / cm^-1^ | Calc. x=1.00  / cm^-1^ | Calc. x=0.98  / cm^-1^ | Calc. Intensity | Vibrational mode  assignment |
| 560 | 554 | 543 | 30 |  |
| 593 | 595 | 583 | 106 | ν C-S δ_sc_ SO_2_ |
| 617 | 614 | 602 | 4 | δ_oop_ ring |
| 603 | 621 | 608 | 2 | δ_oop_ ring |
| 617 | 627 | 615 | 31 | δ_ip_ |
| 734 | 710 | 696 | 264 | ν S-O |
| 752 | 765 | 750 | 78 | δ_oop_ ring |
| 765 | 779 | 763 | 3 | δ_oop_ ring |
| 788 | 795 | 779 | 96 |  |
|  | 811 | 795 | 9 | δ_ip_ ring |
| 808 | 831 | 815 | 117 | δ_oop_ ring |
| 885 | 914 | 896 | 1 | δ_oop_ ring |
| 920 | 941 | 922 | 8 | ν C-S + δ_ip_ ring |
| 937 | 951 | 932 | 0 | δ_oop_ ring |
| 977 | 1000 | 980 | 1 | δ_oop_ ring |
| 1004 | 1017 | 997 | 2 | δ_oop_ ring |
| 1033 | 1035 | 1014 | 32 | δ_ip_ ring |
| 1051 | 1059 | 1038 | 26 | δ_ip_ ring |
| 1079 | 1091 | 1070 | 110 | δ_ip_ ring + ν C-O |
| 1141 | 1165 | 1141 | 313 | δ_ip_ ring + ν_s_ S=O |
| 1164 | 1173 | 1150 | 138 |  |
| 1182 | 1194 | 1170 | 269 | δ_ip_ ring + ν_s_ S=O |
| 1220 | 1238 | 1214 | 36 | δ_ip_ ring |
|  | 1249 | 1224 | 2 | δ_ip_ ring |
| 1270 | 1261 | 1236 | 4 | δ_ip_ ring |
| 1353 | 1358 | 1331 | 370 | ν_as_ S=O |
|  | 1369 | 1342 | 15 | δ_ip_ ring |
| 1417 | 1389 | 1361 | 61 | δ_ip_ ring |
| 1430 | 1445 | 1416 | 21 | δ_ip_ ring |
| 1440 | 1465 | 1435 | 10 | δ_ip_ ring |
| 1459 | 1487 | 1457 | 68 | δ_ip_ ring |
| 1488 | 1521 | 1490 | 61 | δ_ip_ ring |
| 1587 | 1613 | 1581 | 40 | δ_ip_ ring |
|  | 1637 | 1604 | 4 | ν C=C + δ C-H |
| 1639 | 1671 | 1638 | 27 | δ_ip_ ring |

Table S3: Calculated wavenumbers with a correction factor of x=1.00 and x=0.99 and calculated intensity of infrared bands of reduced 1,8-naphthosultone and lithium coordinated to reduced 1,8-naphthosultone.

| **Reduced 1,8-Naphthosultone (B)** | | | **Reduced 1,8-Naphthosultone  + Li ion** | | |  |
| --- | --- | --- | --- | --- | --- | --- |
| Calc. x=1.00  / cm^-1^ | Calc. x=0.99  / cm^-1^ | Calc. Intensity | Calc. x=1.00  / cm^-1^ | Calc. x=0.99  / cm^-1^ | Calc. Intensity | Vibrational mode |
| 555 | 550 | 19 | 556 | 551 | 21 |  |
| 573 | 568 | 2 | 575 | 569 | 23 |  |
| 590 | 584 | 155 | 595 | 589 | 8 |  |
| 596 | 590 | 4 | 604 | 598 | 7 |  |
| 626 | 620 | 39 | 633 | 627 | 18 |  |
| 674 | 667 | 77 | 677 | 670 | 83 |  |
| 685 | 679 | 80 | 717 | 710 | 76 |  |
| 725 | 718 | 162 | 729 | 722 | 144 |  |
| 739 | 731 | 24 | 743 | 736 | 14 |  |
| 776 | 768 | 29 | 783 | 775 | 38 |  |
| 789 | 781 | 98 | 793 | 786 | 90 |  |
| 803 | 795 | 20 | 806 | 798 | 19 |  |
| 852 | 843 | 2 | 864 | 855 | 2 |  |
| 937 | 928 | 7 | 941 | 931 | 8 |  |
| 937 | 928 | 3 | 948 | 938 | 2 |  |
| 942 | 932 | 0 | 952 | 943 | 1 |  |
| 1017 | 1007 | 133 | 1014 | 1003 | 101 |  |
| 1051 | 1041 | 35 | 1055 | 1044 | 30 |  |
| 1061 | 1051 | 26 | 1063 | 1052 | 16 |  |
| 1128 | 1117 | 947 | 1092 | 1081 | 1519 |  |
| 1146 | 1135 | 455 | 1149 | 1137 | 270 |  |
| 1153 | 1142 | 90 | 1154 | 1142 | 10 |  |
| 1197 | 1185 | 57 | 1197 | 1185 | 96 |  |
| 1231 | 1219 | 176 | 1231 | 1218 | 240 |  |
| 1245 | 1233 | 35 | 1237 | 1224 | 111 |  |
| 1270 | 1257 | 427 | 1249 | 1237 | 355 |  |
| 1336 | 1322 | 66 | 1336 | 1323 | 65 |  |
| 1369 | 1355 | 81 | 1365 | 1351 | 66 |  |
| 1411 | 1397 | 140 | 1408 | 1394 | 130 |  |
| 1436 | 1422 | 15 | 1439 | 1424 | 23 |  |
| 1448 | 1434 | 85 | 1453 | 1438 | 62 |  |
| 1474 | 1459 | 52 | 1487 | 1472 | 50 |  |
| 1520 | 1505 | 96 | 1522 | 1507 | 66 |  |
| 1564 | 1549 | 5 | 1575 | 1560 | 6 | ν C=C  + δ_ip_ C-H |
| 1601 | 1585 | 59 | 1607 | 1591 | 53 |  |

Table S4: Calculated wavenumbers with a correction factor of x=1.00 and x=0.99 and calculated intensity of infrared bands of 8-hydrodynaphthalene-1-sulfonate and lithium 8-hydrodynaphthalene-1-sulfonate.

| 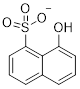 | | | 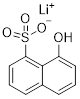 | | |
| --- | --- | --- | --- | --- | --- |
| Calc. x=1.00  / cm^-1^ | Calc. x=0.99  / cm^-1^ | Calc. Intensity | Calc. x=1.00  / cm^-1^ | Calc. x=0.99  / cm^-1^ | Calc. Intensity |
| 554 | 549 | 13 | 555 | 549 | 30 |
| 575 | 569 | 73 | 557 | 552 | 7 |
| 596 | 590 | 82 | 592 | 586 | 117 |
| 633 | 626 | 2 | 635 | 629 | 1 |
| 648 | 641 | 7 | 649 | 642 | 169 |
| 662 | 655 | 175 | 653 | 646 | 22 |
| 769 | 761 | 190 | 769 | 761 | 88 |
| 777 | 770 | 66 | 780 | 773 | 46 |
| 785 | 778 | 17 | 784 | 776 | 30 |
| 791 | 783 | 10 | 790 | 782 | 16 |
| 798 | 791 | 62 | 842 | 834 | 102 |
| 846 | 838 | 67 | 891 | 883 | 25 |
| 910 | 901 | 1 | 915 | 906 | 73 |
| 936 | 927 | 22 | 936 | 927 | 8 |
| 958 | 948 | 0 | 952 | 942 | 0 |
| 997 | 987 | 240 | 992 | 982 | 1 |
| 998 | 988 | 2 | 1010 | 1000 | 3 |
| 1017 | 1007 | 1 | 1016 | 1006 | 154 |
| 1051 | 1040 | 64 | 1051 | 1040 | 73 |
| 1100 | 1089 | 266 | 1107 | 1096 | 8 |
| 1119 | 1108 | 163 | 1123 | 1112 | 82 |
| 1124 | 1112 | 100 | 1161 | 1149 | 379 |
| 1170 | 1159 | 20 | 1169 | 1157 | 140 |
| 1183 | 1172 | 3 | 1181 | 1170 | 6 |
| 1212 | 1200 | 67 | 1203 | 1191 | 79 |
| 1224 | 1212 | 20 | 1224 | 1212 | 14 |
| 1273 | 1260 | 383 | 1240 | 1228 | 432 |
| 1291 | 1278 | 207 | 1302 | 1289 | 188 |
| 1327 | 1313 | 26 | 1329 | 1316 | 25 |
| 1364 | 1351 | 51 | 1363 | 1349 | 51 |
| 1381 | 1367 | 84 | 1378 | 1364 | 91 |
| 1431 | 1417 | 95 | 1448 | 1433 | 97 |
| 1475 | 1461 | 34 | 1471 | 1456 | 138 |
| 1476 | 1462 | 97 | 1499 | 1484 | 28 |
| 1540 | 1524 | 67 | 1543 | 1528 | 93 |
| 1605 | 1589 | 62 | 1604 | 1588 | 92 |
| 1635 | 1602 | 13 | 1636 | 1603 | 19 |
| 1650 | 1617 | 29 | 1651 | 1618 | 18 |


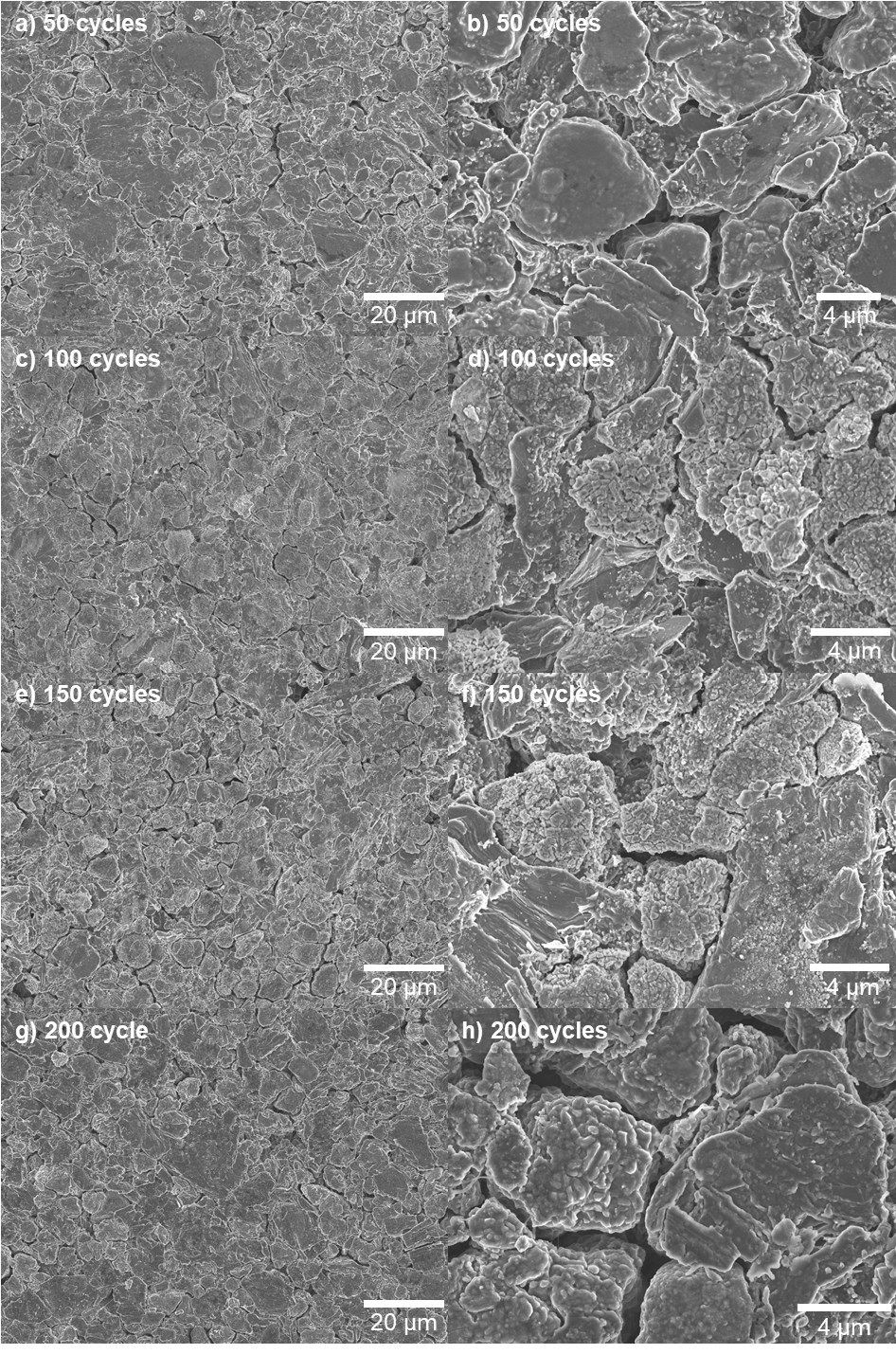


Figure S16: SEM images of AG + 20 % SiO_x_ anodes charge/discharge cycled for a-b) 50 cycles, c-d) 100 cycles, e-f) 150 cycles, and g-h) 200 cycles.


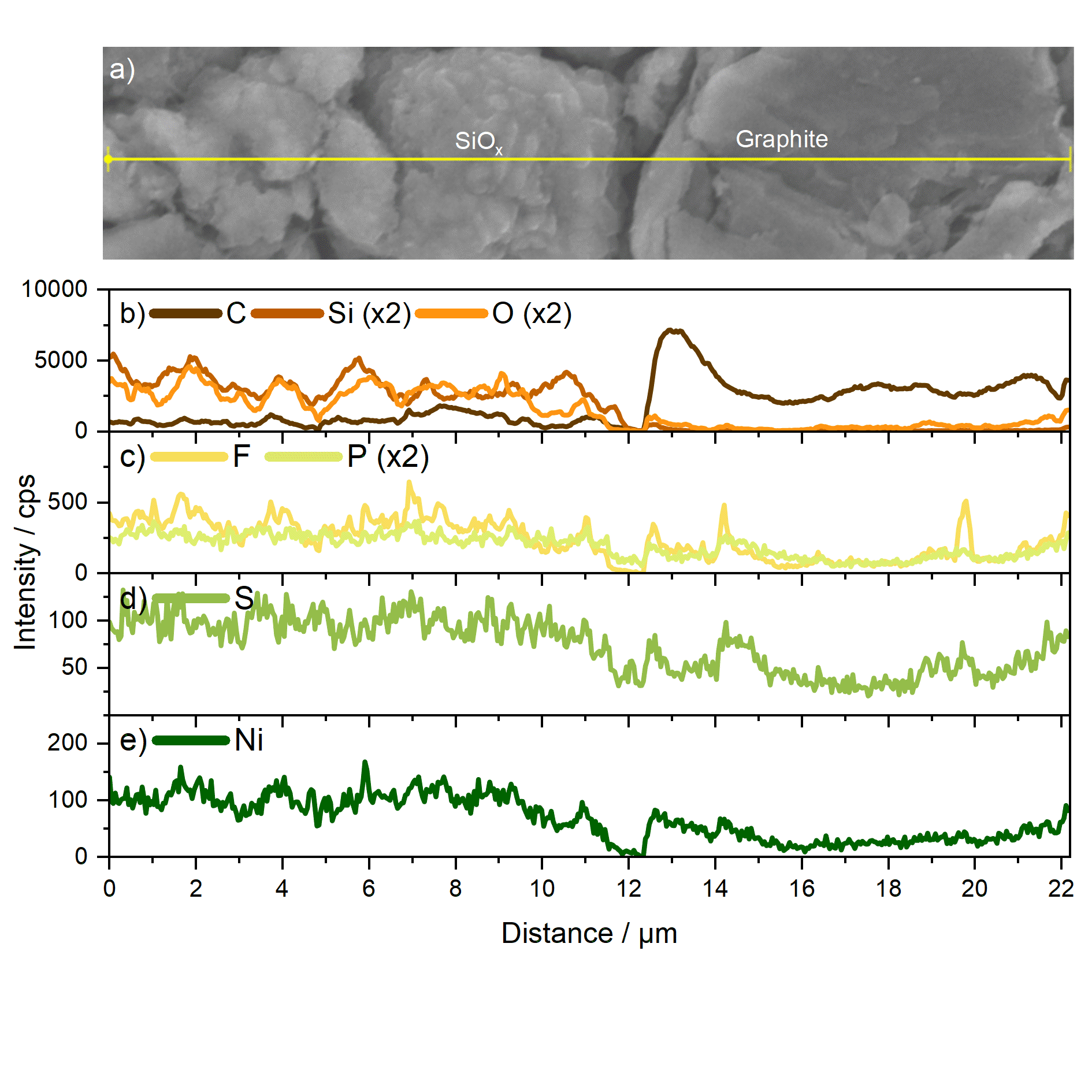


Figure S17: a) SEM image of the anode from NMC811‖ AG+20 % SiO_x_ multilayer pouch cells charge/discharge cycled to 200 cycles showing the EDX line scan on a SiO_x_ particle and an AG particle. EDX line scan intensities of b) C, Si (x2), O (x2), c) F, P (x2), d) S, and e) Ni on the particles shown in a). The electrodes were washed with DMC before the measurement.
